# Absence of S100A4 in the mouse lens induces an aberrant retina-specific differentiation program and cataract

**DOI:** 10.1038/s41598-021-81611-y

**Published:** 2021-01-26

**Authors:** Rupalatha Maddala, Junyuan Gao, Richard T. Mathias, Tylor R. Lewis, Vadim Y. Arshavsky, Adriana Levine, Jonathan M. Backer, Anne R. Bresnick, Ponugoti V. Rao

**Affiliations:** 1 Department of Ophthalmology, Duke University School of Medicine, Durham, NC USA; 2 Department of Physiology and Biophysics, Stony Brook University, Stony-Brook, NY USA; 3 Department of Pharmacology and Cancer Biology, Duke University School of Medicine, Durham, NC USA; 4 Department of Biochemistry, Albert Einstein College of Medicine, Bronx, NY USA; 5 Department of Molecular Pharmacology, Albert Einstein College of Medicine, Bronx, NY USA

**Keywords:** Developmental biology, Diseases

## Abstract

S100A4, a member of the S100 family of multifunctional calcium-binding proteins, participates in several physiological and pathological processes. In this study, we demonstrate that S100A4 expression is robustly induced in differentiating fiber cells of the ocular lens and that *S100A4* ^(−/−)^ knockout mice develop late-onset cortical cataracts. Transcriptome profiling of lenses from *S100A4* ^(−/−)^ mice revealed a robust increase in the expression of multiple photoreceptor- and Müller glia-specific genes, as well as the olfactory sensory neuron-specific gene, *S100A5*. This aberrant transcriptional profile is characterized by corresponding increases in the levels of proteins encoded by the aberrantly upregulated genes. Ingenuity pathway network and curated pathway analyses of differentially expressed genes in *S100A4* ^(−/−)^ lenses identified Crx and Nrl transcription factors as the most significant upstream regulators, and revealed that many of the upregulated genes possess promoters containing a high-density of CpG islands bearing trimethylation marks at histone H3K27 and/or H3K4, respectively. In support of this finding, we further documented that *S100A4* ^(−/−)^ knockout lenses have altered levels of trimethylated H3K27 and H3K4. Taken together, our findings suggest that S100A4 suppresses the expression of retinal genes during lens differentiation plausibly via a mechanism involving changes in histone methylation.

## Introduction

The developing eye represents a fascinating model to interrogate the molecular mechanisms governing the commitment and specification of various ocular tissues, including the retina and lens. During embryogenesis, the retina develops from the neural epithelium of the optic vesicle, whereas the lens develops from the lens vesicle (LV) which is formed upon invagination of the lens placode ectoderm followed by separation of the LV from the surface ectoderm^1,2^. Once the LV is formed, the posterior cells of the LV elongate and differentiate into lens primary fibers, while the anterior LV becomes the epithelium covering the anterior portion of the lens. After the primary fibers fill the LV, epithelial cells at the equator exit the cell cycle and differentiate into secondary fibers. These fibers undergo terminal differentiation and eventually lose all cellular organelles, including the nuclei, and assume a radial packing organization with perfect hexagonal symmetry. The lens expresses various tissue-preferred and tissue-specific proteins including crystallins, aquaporin-0, gap junctional proteins and beaded filament cytoskeletal proteins required for maintaining lens function^1^.

Lens morphogenesis and growth are governed by various transcription factors (e.g. Pax6, Sox2, Six3, Maf, Prox1 and Hsf4) and growth factor regulated signaling pathways, including the TGF-β/BMP, FGF, Wnt, integrin, sonic hedgehog and Notch pathways. However, little is known about gene suppressive mechanisms and their role in lineage determination of the lens^1^. Our current data suggest that one protein potentially involved in these suppressive mechanisms is S100A4, a member of the large S100 family of calcium binding proteins^3,4^, whose expression is tissue-, cell- and cell cycle-specific^3,5^.

S100A4, used as a prognostic cancer biomarker^3^, has been shown to participate in proliferation, differentiation, cell motility, invasion, and immunity^3,4,6–8^. This protein interacts with several intracellular targets, including actin and non-muscle myosin IIA^9^, and is localized predominantly in the cytosol with some additional distribution to the nucleus^3,10^. S100A4 can regulate the signaling activity of various growth factors^11,12^, the secretion of matrix metalloproteinases^13^, and the epithelial to mesenchymal transition^3,7^. S100A4 expression is regulated transcriptionally by the Wnt/β-catenin pathway^14^, and epigenetically by DNA methylation^15^. Similar to other S100 proteins, Ca^2+^ binding to S100A4 promotes its interaction with target proteins^16^.

In this study, we found that S100A4 localizes specifically to mouse (*Mus musculus*) lens fiber cells, with expression being robustly upregulated in differentiating primary and secondary lens fibers. We further demonstrated that S100A4 knockout mice develop late-onset lens cortical opacification. To understand the role of S100A4 in lens differentiation and function, we evaluated the global gene expression profile of the S100A4 knockout lens using RNA-seq. This analysis revealed that, without S100A4, lens fibers exhibit a robust upregulation of the retinal transcriptome, particularly photoreceptor-specific genes and transcription factors required for specifications of rods, cones and Müller glia. These transcriptome changes are accompanied by chromatin remodeling involving histone-3 lysine (H3K) methylation. Taken together, our findings suggest that S100A4 suppresses the transcription of sensory neuron genes during lens development and differentiation.

## Results

### S100A4 is robustly expressed in and distributes discretely to differentiating and mature lens fibers

**Figure 1:**
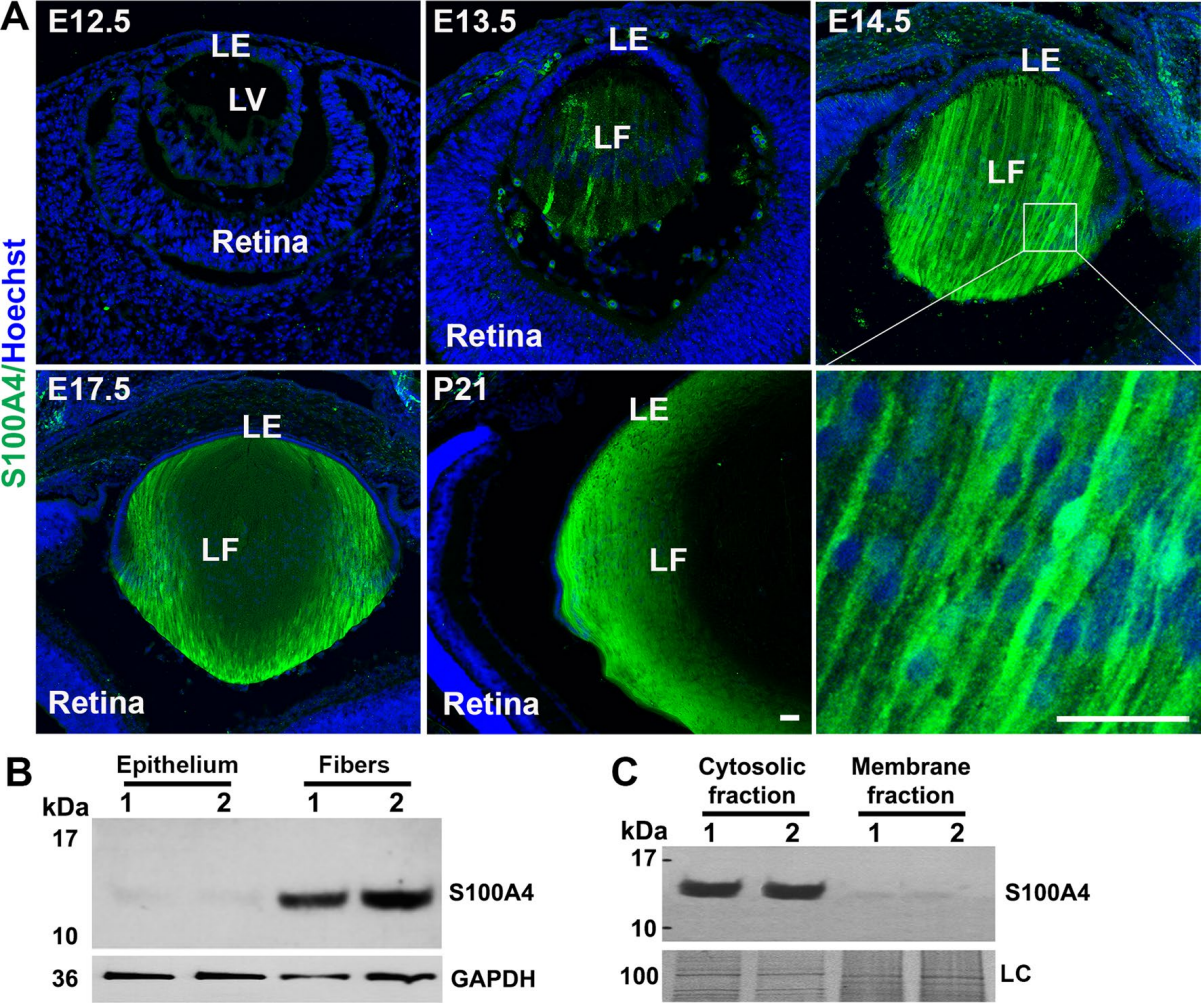
Expression and distribution of S100A4 in differentiating and mature fibers of the mouse lens. (**A**) Immunofluorescent staining of S100A4 distribution (green) in sagittal sections of embryonic (E12.5, 13.5, 14.5 and 17.5) and differentiated (P21) mouse lenses. Nuclei are stained with Hoechst (blue). There seems to be no nuclear localization of S100A4 in lens fibers as shown in the bottom right panel (magnified image of the indicated area of E14.5 lens). *LE* lens epithelium, *LV* lens vesicle, *LF* lens fibers. Scale bars 20 μm. Representative images of one of three independent specimens analyzed are shown. (**B**) Immunoblot analysis of S100A4 in P21 mouse lens epithelium and fiber mass reveals its distribution specifically in fibers. (**C**) Immunoblot of S100A4 in P21 mouse lens lysates show its distribution primarily to the soluble (100,000 g supernatant) fractions compared to membrane fractions (100,000 g pellet). For soluble and membrane fractions, protein samples from the respective fractions separated on SDS-PAGE were stained with Gelcode blue with the staining intensity of the indicated protein band used as a loading control (LC). GAPDH was used as a loading control (**B**). Lanes 1 and 2 represent two biological replicates (**B**,**C**).

The expression pattern of S100A4 in developing mouse lens was assessed by immunofluorescence staining of eye sections obtained from embryonic, neonatal and weaning stage animals. Figure 1A shows the marked upregulation of S100A4 expression in differentiating and elongating primary fibers of the embryonic lens, starting at E12.5 and continuing through weaning age (analyzed up to P21). Notably, little to no S100A4 was found in the lens epithelium and none was observed in the retina, cornea or other ocular tissues. Despite the previously published suggestion that S100A4 may be expressed in the bovine retina^17^, we did not detect S100A4 in mouse retina. Whether this discrepancy is related to a species-specific difference requires further evaluation in a future study.

Consistent with immunofluorescence results, S100A4 was detected only in lens fiber cell fraction with almost no S100A4 detected in the epithelial fraction (Fig. 1B). At the subcellular level, S100A4 exhibits a cytoplasmic distribution pattern with no obvious presence in the nucleus of lens fiber cells (Fig. 1A, bottom right panel). Additionally, to determine whether S100A4 is present in the nuclei of lens fibers, the nuclear fraction was extracted from P30 lens fibers and subjected to immunoblotting. Whereas some S100A4 was detected in the nuclear fraction, its relative amount did not exceed that of contaminating GAPDH used as a cytosolic marker (Supplementary Fig. S1A), which suggests that S100A4 is essentially a cytosolic protein in the lens. Immunoblot analysis further confirmed that S100A4 is a soluble protein with only a trace amount detected in the membrane-enriched fraction (Fig. 1C). Because S100 proteins can be secreted and interact with various cell surface targets to regulate cellular activities^9^, we tested whether S100A4 could be secreted from the lens. Lenses from P22 old mice were organ cultured for 48 h and conditioned medium was concentrated and subjected to immunoblotting. However, no detectable S100A4 was observed (Supplementary Fig. S1B).

Additionally, expression of *S100A4* and several other members of the S100 family, was reliably detected in RNA-seq analyses of P30 mouse lenses (Supplementary Table S1). The relative levels of *S100A6* expression (relative units-RU: 23,779) was found to be higher than that of *S100A4* (14,401 RU), while the expression levels of *S100A10* (4,056 RU), *S100A11* (1,155 RU), *S100A16* (923 RU), *S100A1* (189 RU), *S100A13* (39 RU) and *S100B* (23 RU) were much lower. Consistent with our findings, the iSyTE database for the gene expression profile of mouse lens also confirms expression of various genes of the S100 family^18^.

### S100A4 deletion induces late-onset lens opacification in mice

**Figure 2:**
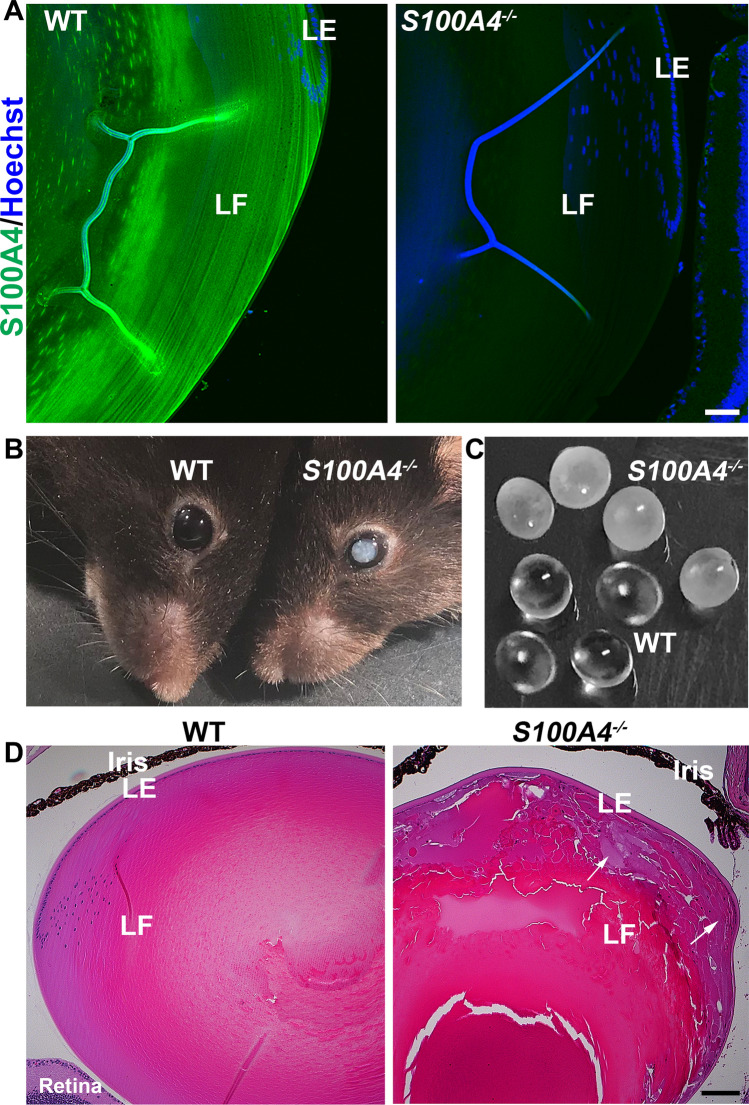
S100A4 deletion induces late-onset opalescent cataract in the mouse. (**A**) Confirmation of S100A4 absence in P30 *S100A4*^−/−^ mouse lens relative to WT littermate lenses based on immunofluorescence analysis. Scale bar 50 µm. (**B**) A 9-month-old S*100A4*^−/−^ mouse exhibits an opalescent cataract phenotype compared to the clear lens in a littermate WT mouse. (**C**) Enucleated lenses from 9-month-old *S100A4*^−/−^ mice show opalescent opacity compared to clear lenses of WT mice. (**D**) Histological analysis (hematoxylin and eosin staining) of the lens from 8.5 months-old *S100A4*^−/−^ mouse reveals extensive swelling and disorganization of cortical fibers (indicated with arrows). Representative images are taken from three biological replicates. *LE* lens epithelium, *LF* lens fibers. Scale bar 100 μm.

Having found that S100A4 is robustly expressed in the wild type (WT) mouse lens, we asked whether S100A4 is required for lens development and function. We examined the ocular phenotype of the well-characterized *S100A4*^−/−^ mouse, which has been shown to grow and breed normally^19^. We confirmed absence of S100A4 expression in *S100A4 *^−/−^ mouse lenses by immunofluorescence analysis (Fig. 2A). These mice displayed bilateral opalescent cataract with onset at ~ 8 months of age (Fig. 2B,C), with the phenotype being much more prevalent in female animals. While there was no difference in lens weight between *S100A4*^−/−^ (7.15 ± 0.08 mg/lens; n = 10) and WT (7.11 ± 0.04 mg/lens; n = 8) mice, histological analysis of *S100A4*^−/−^ mouse cataractous lenses revealed swollen and disorganized fibers at the outer cortical region exhibiting abnormal accumulation of vacuoles, relative to those in the nuclear region (Fig. 2D). Additionally, none of the lenses from the *S100A4*^−/−^ mice exhibited a nuclear cataract as shown in Fig. 2C. These data demonstrate that S100A4 is required for maintenance of lens transparency. Further studies are required to understand the molecular basis of cataract formation and apparent gender-related difference in cataract formation in *S100A4*^−/−^ mice.

### S100A4^−/−^ mouse lens maintains normal actin cytoskeleton organization, calcium level, and gap junctional activity

We next explored whether S100A4 plays a role in actin cytoskeleton organization, calcium homeostasis, and gap junctional activity, all of which are key determinants of lens transparency^20,21^. These studies did not uncover any significant differences between mature lenses of *S100A4*^−/−^ and littermate WT mice, either in actin cytoskeletal organization (1 month-old mice; Supplementary Fig. S2A), levels of calcium (2 month-old mice, Supplementary Fig. S2B), and gap junctional coupling activity (2 month-old mice, Supplementary Fig. S2C), suggesting that S100A4 does not influence any of these important characteristics in the mature lens. Whether these characteristics are altered as a function of aging in the *S100A4*^−/−^ mouse lens needs to be addressed in future studies.

### S100A4 deletion induces aberrant gene expression in the mouse lens

**Figure 3:**
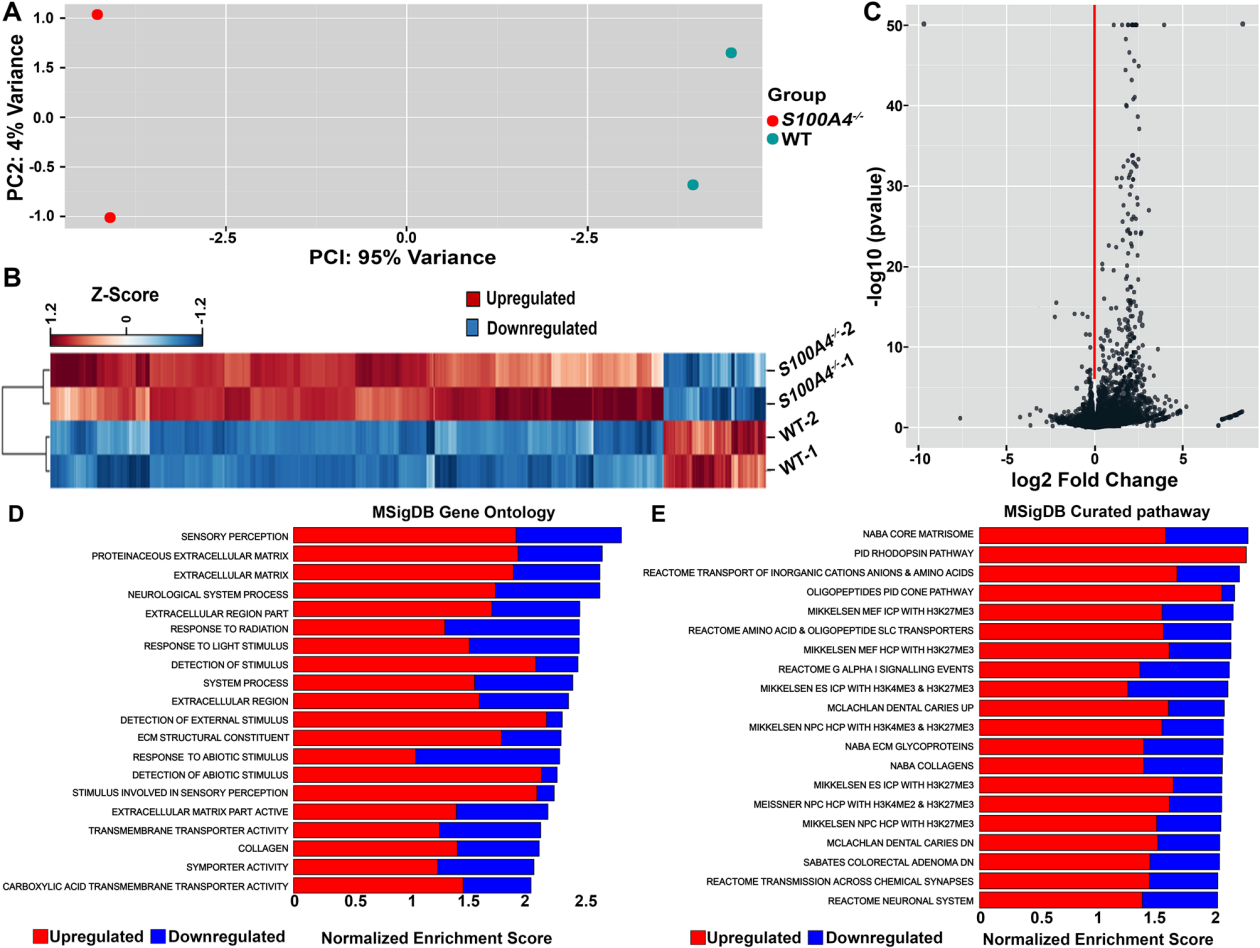
Aberrant profile of the S100A4 knockout lens transcriptome. (**A**) Two-dimensional plot of the principal component (PC) analysis for RNA-seq data of *S100A4*^−/−^ (red) and littermate WT (green) mouse lenses (n = 2; for each sample 6–12 lenses were pooled derived from P30 mice. The X axis and Y axis depict the PC1 and PC2, respectively. The value after the PC identifier displays the proportion of variance. (**B**) Heat map of expression values of differentially expressed genes in *S100A4*^−/−^ lenses compared to littermate WT lenses. Only differentially expressed genes (≥ twofold difference in expression, adjusted P value of ≤ 0.05) were used for creating the heat map. Each column corresponds to one sample, and each row corresponds to a gene. The genes are hierarchically clustered using a correlation distance with complete linkage and show the similarity of their expression profiles between *S100A4*^−/−^ and littermate WT samples. (**C**) The volcano plot showcasing the results of the comparison between S100A4^−/−^ and littermate WT samples. The log2-fold changes which were calculated based on *S100A4*^−/−^ /WT were plotted on the x-axis. The − log10 p-values are plotted on the y axis. Each dot represents a gene. (**D**,**E**) Gene enrichment analysis of differentially expressed genes in *S100A4*^−/−^ mouse lens. Gene enrichment analysis was performed using the Molecular Signatures Database (MSigDB) using the results of the comparison of genes in *S100A4*^−/−^ lenses compared to WT lenses. The top 20 most significantly enriched GO terms (**D**) and KEGG curated pathways (**E**) were illustrated by their normalized enrichment score. Red and blue bars show proportional upregulated and downregulated genes in a given pathway, respectively.

To gain insight into the role of S100A4 in lens function, we analyzed gene expression profiles of 1-month-old *S100A4*^−/−^ and their WT littermates using RNA-seq. Two biological replicates (derived from 6 to 12 pooled lenses/sample) were used per group. The total number of expressed genes was 14,021 in WT (Supplementary Table S1) and 14,925 in *S100A4*^−/−^ (Supplementary Table S1) lenses. Principal component analysis revealed that the greatest contributor to variance of individual gene counts (by 95%) was the genotype (Fig. 3A). The variation between biological replicates of the same group was much smaller (by 4%) (Fig. 3A). Consistently, heat map construction (z-score normalized) and hierarchical clustering of the differentially expressed genes (DEGs; which we define as genes exhibiting a fold-change ≥ 2 and an adjusted p-value ≤ 0.05) revealed a closer clustering between the two *S100A4*^−/−^ and two WT biological replicates than among *S100A4*^−/−^ and WT transcriptomes (Fig. 3B).

This analysis revealed a dramatic upregulation of several DEGs in *S100A4*^−/−^ lenses (Fig. 3B). A volcano plot in Fig. 3C demonstrates that the number of upregulated genes in the knockout was much higher than the number of downregulated genes. Based on the criteria of fold-change ≥ 2 and adjusted p-value of ≤ 0.05, 383 unique transcripts were upregulated while 5 gene transcripts exhibited downregulation in *S100A4*^−/−^ lenses (Supplementary Table S2). The fact that ~ 99% of DEGs were upregulated suggests that S100A4 functions to suppress gene expression in the lens. All raw RNA-seq data and normalized expression values are available in the Gene Expression Omnibus with accession number- GSE143909.

Gene Set Enrichment Analysis (GSEA) including gene ontology (Fig. 3D) and curated pathways from MSigDB (containing KEGG and Reactome data) (Fig. 3E), revealed that the genes upregulated in *S100A4*^−/−^ lenses encode proteins involved in sensory perception, neuronal system processes, rod and cone phototransduction pathways, transport of amino acids, G protein signaling, synaptic transmission and matrisome, and that the promoters of several upregulated genes contained a high density of CpG islands bearing trimethylation marks at histone 3-lysine (H3K27 and H3K4). Figure 3D,E depict the proportion of upregulated vs. downregulated genes within the indicated pathways.

Downregulated genes in *S100A4*^−/−^ lenses included *Rab4a, Pttg1, Cck* and *Dynlt1b* (Supplementary Table S2). GSEA did not reveal significant gene enrichment for any of the downregulated genes in *S100A4*^−/−^ lenses.

### Robust induction of the olfactory sensory neuron gene S100A5 in S100A4^−/−^ lenses

**Figure 4:**
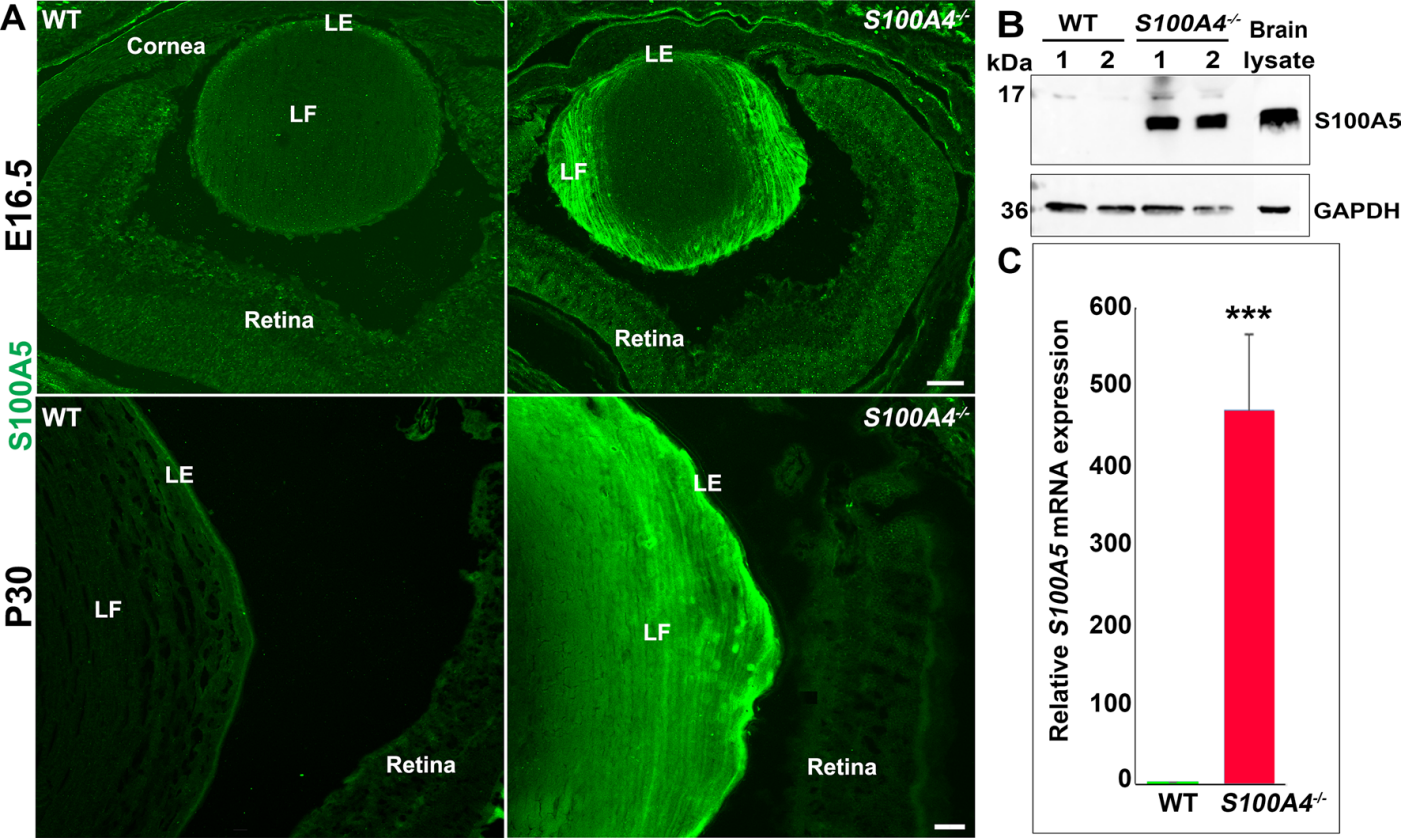
Robust induction of S100A5 gene expression and protein levels in mouse lenses lacking S100A4. (**A**) Immunofluorescence analysis of *S100A4*^−/−^ lens sagittal cryofixed sections derived from the E16.5 and P30 mice reveals induction of S100A5 protein, distributing (bright green stain) discretely to the fiber cells in comparison to its absence in littermate WT lenses. Similar to S100A4 (Fig. 1), S100A5 is not detectable in non-lens tissues of the eye. Data represent results from three biological replicates. *LE* lens epithelium, *LF* lens fibers. Scale bars 50 and 20 μm for E16.5 and P30 specimens, respectively. (**B**) Immunoblotting analysis based confirmation of increased levels of S100A5 in P30 *S100A4*^−/−^ lenses relative to littermate WT lenses. Data were shown for two representative biological replicates (lanes 1, 2), a total of four biological replicates were analyzed. GAPDH was used as a loading control. Brain lysate (in panel **B**) derived from P30 mice was used as a positive control. (**C**) Robust and significant (***P > 0.001, Student t test; n = 8) upregulation of S100A5 expression in P30 *S100A4*^−/−^ lenses compared to littermate WT lenses was confirmed by qRT-PCR and presented as relative-fold change in expression compared to WT.

Interestingly, the gene with the highest level of induction in the *S100A4*^−/−^ lens was *S100A5* (~ 275-fold) (Supplementary Table S1). S100A5 is normally expressed in distinct brain regions^22^, most notably the olfactory bulb and olfactory sensory neurons^23^, where it is thought to participate in olfactory signaling^24^. We confirmed increased expression of the S100A5 protein in *S100A4*^−/−^ lenses by immunostaining and Western blotting analysis. Similar to S100A4 in the normal lens, S100A5 localizes primarily to lens fiber cells (Fig. 4A). Induction of S100A5 expression in the S100A4 knockout lens was observed starting at E16.5 (Fig. 4A). Analysis of P30 (1-month-old) lenses derived from *S100A4*^−/−^ mice also confirmed a robust increase in S100A5 protein levels by immunofluorescence (Fig. 4A), immunoblotting analyses (Fig. 4B), and qRT-PCR analyses (Fig. 4C) with no signal detected in littermate WT lenses. Neither S100A4 nor S100A5 were detected by either technique in the retinas of WT and *S100A4*^−/−^ mice (Supplementary Fig. S3), while lens lysate samples from WT and *S100A4*^−/−^ mice showed positive bands for S100A4 and S100A5, respectively (Supplementary Fig. S3). S100A5 is one of the least studied members of the S100 family^25^.

### Upregulation of retina-specific genes in S100A4^−/−^ lenses

In addition to *S100A5*, the most significantly upregulated genes in *S100A4*^−/−^ lenses encode transcription factors regulating retinal and photoreceptor development (*Nrl*, *Crx*, *Nr2e3*, *Rax*, *Vsx2*, *Otx1* and *2*, *neurod1 and Rorb)* as well as proteins regulating retinogenesis (*Mab21l2*) and performing various photoreceptor-specific functions^26,27^. The latter included rhodopsin (*Rho*), transducin (*Gngt1* and *Gnat2*), peripherin-2 (*Prph2*), Rom1 (*Rom1*), arrestin-1 (*Sag*)*,* rhodopsin kinase (*Grk1*), cGMP phosphodiesterase (*Pde6 a, b* and *g*)*,* recoverin (*Rcvrn*), guanylate cyclase (*Gucy2F*), complexins (*Cplx*) and others (Table 1 and Table S2).

**Table 1:** Upregulated expression of retinal genes in the *S100A4*^−/−^ mouse lens.

Gene	Fold change	P value	Adj P value
*Aipl1*	3.9	5.96E−13	7.67E−11
*Best2*	4.5	1.54E−31	5.10E−29
*Cacna1g*	2.6	3.33E−08	2.67E−06
*Cnga1*	3.9	1.58E−11	1.77E−09
*Cplx3*	4.3	6.40E−06	3.539E−4
*Cplx4*	4.6	5.75E−09	5.06E−07
*Crx*	4.0	3.18E−18	6.00E−16
*Esrrb*	2.0	1.79E−11	1.98E−09
*Gnat1*	4.4	1.14E−163	3.41E−160
*Gngt1*	4.2	3.64E−18	6.79E−16
*Grk1*	4.8	9.22E−42	5.50E−39
*Guca1a*	3.9	2.75E−12	3.31E−10
*Guca1b*	4.0	1.13E−29	3.51E−27
*Lhx2*	2.0	4.41E−09	3.94E−07
*Mab21l2*	3.4	3.73E−13	4.88E−11
*Neurod1*	4.2	2.83E−07	1.99E−05
*Nr2e3*	2.8	1.74E−07	1.26E−05
*Nrl*	4.2	1.66E−32	6.33E−30
*Otx1*	5.0	1.83E−06	1.14E−4
*Otx2*	3.8	7.15E−05	3.006E−3
*Pdc*	5.4	2.40E−39	1.24E−36
*Pde6a*	3.5	1.22E−40	6.48E−38
*Pde6b*	3.0	5.23E−24	1.20E−21
*Pde6g*	3.8	9.25E−32	3.37E−29
*Prom1*	4.1	1.49E−14	2.20E−12
*Prph2*	4.3	5.63E−82	1.05E−78
*Rax*	4.3	3.51E−12	4.16E−10
*Rbp3*	5.1	2.67E−128	6.65E−125
*Rcvrn*	5.2	3.00E−29	9.15E−27
*Rdh12*	2.8	4.23E−06	2.392E−4
*RGS9*	2.6	3.13E−09	2.84E−07
*Rho*	4.5	4.94E−238	2.46E−234
*Rom1*	2.7	1.22E−30	3.86E−28
*Rorb*	3.5	2.78E−06	1.612E−4
*Rp1*	5.7	7.99E−38	3.98E−35
*Rs1*	4.8	1.95E−33	8.07E−31
*Sag*	3.4	5.65E−49	4.44E−46
*Six6*	5.6	7.87E−08	5.87E−06
*Syp*	3.7	2.27E−22	4.83E−20
*Tub*	4.8	3.20E−13	4.23E−11
*Tulp1*	4.0	7.93E−25	1.88E−22
*Vsx2*	4.3	3.05E−23	6.69E−21

**Figure 5:**
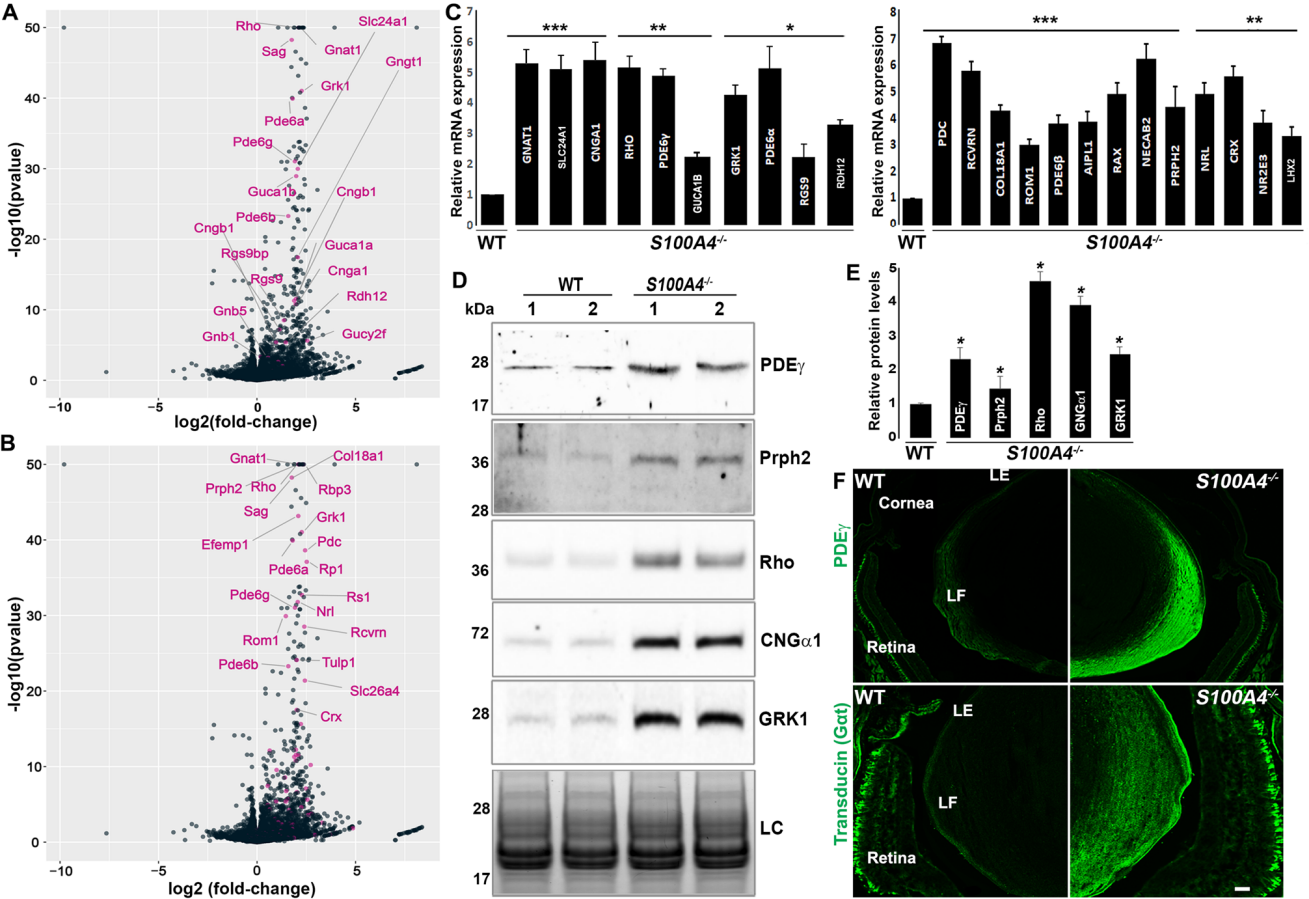
Aberrant induction of photoreceptor and retina-specific genes in *S100A4*^−/−^ mouse lenses. (**A**,**B**) Volcano plots highlighting the upregulation of selected photoreceptor and retinal genes in *S100A4*^−/−^ lenses (labeled in pink), identified based on gene set enrichment by curated pathways and GO terms, respectively. (**C**) qRT-PCR confirmation of significant upregulation of the indicated photoreceptor and retina specific genes in *S100A4*^−/−^ mouse lenses relative to littermate WT lenses (***P < 0.001, **P < 0.01 and *P < 0.05, based on Student’s t test and an n = 4 pooled samples consisting of 6–10 lenses each). (**D**,**E**) Immunoblotting based confirmation of increased levels of selected photoreceptor specific proteins in *S100A4*^−/−^ lens lysates (100,000 g pellet) compared to WT lenses. Lanes 1, 2 represent two biological replicates. *LC* loading control (lens insoluble fractions resolved by SDS–PAGE, stained with Gelcode blue, and staining intensity of the indicated protein band used for normalization). *P < 0.05, based on Student’s t test and n = 4. (**F**) Representative images (from three biological replicates) from immunofluorescence based confirmation of upregulation of PDEγ and transducin in *S100A4*^−/−^ mouse lenses compared to WT lenses. *LE* lens epithelium, *LF* lens fibers; scale bar 20 μm.

Accordingly, the GSEA analysis revealed an enrichment of genes belonging to the rod and cone phototransduction, “G-alpha1 signaling”, “neuronal system”, “transmission across chemical synapse”, “sensory perception”, “neurological system process” and “response to light stimulus” pathways (Fig. 3D). Figure 5A,B volcano plots showcase the DEGs involved in the phototransduction pathway and sensory perception, respectively. The upregulation of several retina and photoreceptor-specific genes in *S100A4*^−/−^ lenses was confirmed by qRT-PCR (Fig. 5C). Additionally, immunoblot and immunofluorescence analyses showed increased levels of several proteins encoded by these genes in *S100A4*^−/−^ lenses (Fig. 5D–F). Having recorded these results in P30 lenses, we also asked whether upregulation of some of these retinal genes begins earlier at the neonatal stage. Using qRT-PCR analysis of RNA derived from pooled *S100A4*^−/−^ lens samples, we confirmed a significant increase in expression of selected genes including *Nrl*, *Otx2, Rho, Rcvrn* and *Slc61a* in P1 mice (Supplementary Fig. S4). Taken together, these results indicate an aberrant induction of retina-specific genes in *S100A4*^−/−^ lenses.

Furthermore, to identify upstream regulators involved in altered gene expression in *S100A4*^−/−^ lenses, we performed network analysis to determine enrichment of downstream genes using the Qiagen Ingenuity Pathway Analysis (IPA) tool. This analysis identified Crx, Rho and Nrl as the top three, most significant upstream regulators (Supplementary Table S3). Crx and Nrl are known crucial transcriptional regulators of photoreceptor development and differentiation^27,28^, while Rho is a key light-sensitive receptor involved in visual phototransduction^29^. Of note, induction of photoreceptor-specific differentiation programs has been similarly reported in medulloblastoma under the control of transcription factors Nrl and Crx^30^. Interestingly, the deficiency of Notch2 and E2F has also been found to be associated with upregulation of certain retinal genes in the mouse lens^18^.

*S100A4*^−/−^ lenses also displayed an induction of genes encoding various extracellular matrix glycoproteins and collagens, including reelin, interphotoreceptor matrix proteoglycans, *Efemp1*, fibulins, endostatin producing collagen XVIII (*Col18a1*) and several others (Supplementary Table S2 and Supplementary Fig. S5A). It is noteworthy that overexpression of endostatin producing collagen XVIII has been previously reported to induce cataracts in transgenic mice^31^. RT-PCR (Supplementary Fig. S5B), immunoblot (Supplementary Fig. S5C,D) and qRT-PCR (Supplementary Fig. S5E) confirmed a significant increase in the levels of collagen XVIII in *S100A4*^−/−^ lenses relative to WT controls. Therefore, it is plausible that altered collagen XVIII expression in the *S100A4*^−/−^ lens is at least partially responsible for the observed lens opacification.

### Upregulation of genes responsible for Müller cell differentiation, glutamate metabolism and solute transport in the S100A4^−/−^ lens

The transcriptome of *S100A4*^−/−^ lenses displayed an upregulation of two transcription factors *Lhx2* and *Plagl1*, required for Müller cell specification and differentiation^32,33^. These glial cells serve to support retinal neurons and play a crucial role in maintaining the structural and functional stability of the retina^34^. Other Müller cell-specific or abundant genes upregulated in the *S100A4*^−/−^ lens included glutamine synthetase (*Glul*), retinaldehyde binding protein 1 (*Rlbp1*), glutamate transporters (*Slc1a3, Slc17a7, Scl17a8*), GABA transporters (*Slc6a13, Slc6a1, Slc6a6, Slc6a9*), glutamate receptor (*Gria3)* and glutamate decarboxylase (*Gad1*) (Supplementary Table S2; Fig. S6A). We independently confirmed the increased expression of glutamine synthetase (*Glul*) by RT-PCR (Supplementary Fig. S6B), qRT-PCR (Supplementary Fig. S6C) and immunoblotting (Supplementary Fig. S6D, E). These results together with upregulated expression of photoreceptor specific genes in *S100A4*^−/−^ mouse lens reveal an aberrant induction of retinal differentiation programs in the lens in the absence of S100A4.

*S100A4*^−/−^ lenses also exhibited upregulated expression of several solute carrier transport genes including *Slc4a10, Slc4a1, Slc16a2, Slc12a5, Slc26a4, Slc24a1, Slc7a1, Slc38a8, Slc5a5, Slc22a8, Slc4a5, Slc13a4* and other transporters (Supplementary Table S2; Fig. S7A, B). We not only confirmed increased expression of Slc6a6 which encodes the taurine transporter (TauT) in *S100A4*^−/−^ lenses (Supplementary Fig. S7B), but also found that levels of this protein were markedly elevated in *S100A4*^−/−^ lenses (Supplementary Fig. S7C, D). Taurine is a well-characterized osmolyte and has been shown to influence S100A4 expression^35^.

Interestingly, lenses of older but not young *S100A4*^−/−^ mice develop cataracts characterized by swelling of lens cortical fibers (Fig. 2). It appears that the presumed osmotic changes resulting from dysregulated expression of various solute carrier transport genes identified above including glutamate, GABA, taurine and others, might not per se lead to cataract formation in young *S100A4*^−/−^ mice, since dysregulation of solute carrier and extracellular matrix gene expression was evident in P30 *S100A4*^−/−^ mice. However, the effects of these changes are likely exacerbated by age-related stress factors, leading to cataract development in older *S100A4*^−/−^ mouse lenses.

### Expression of non-lineage genes in the S100A4^−/−^ lens is associated with changes in H3K27 and H3K4 trimethylation

**Figure 6:**
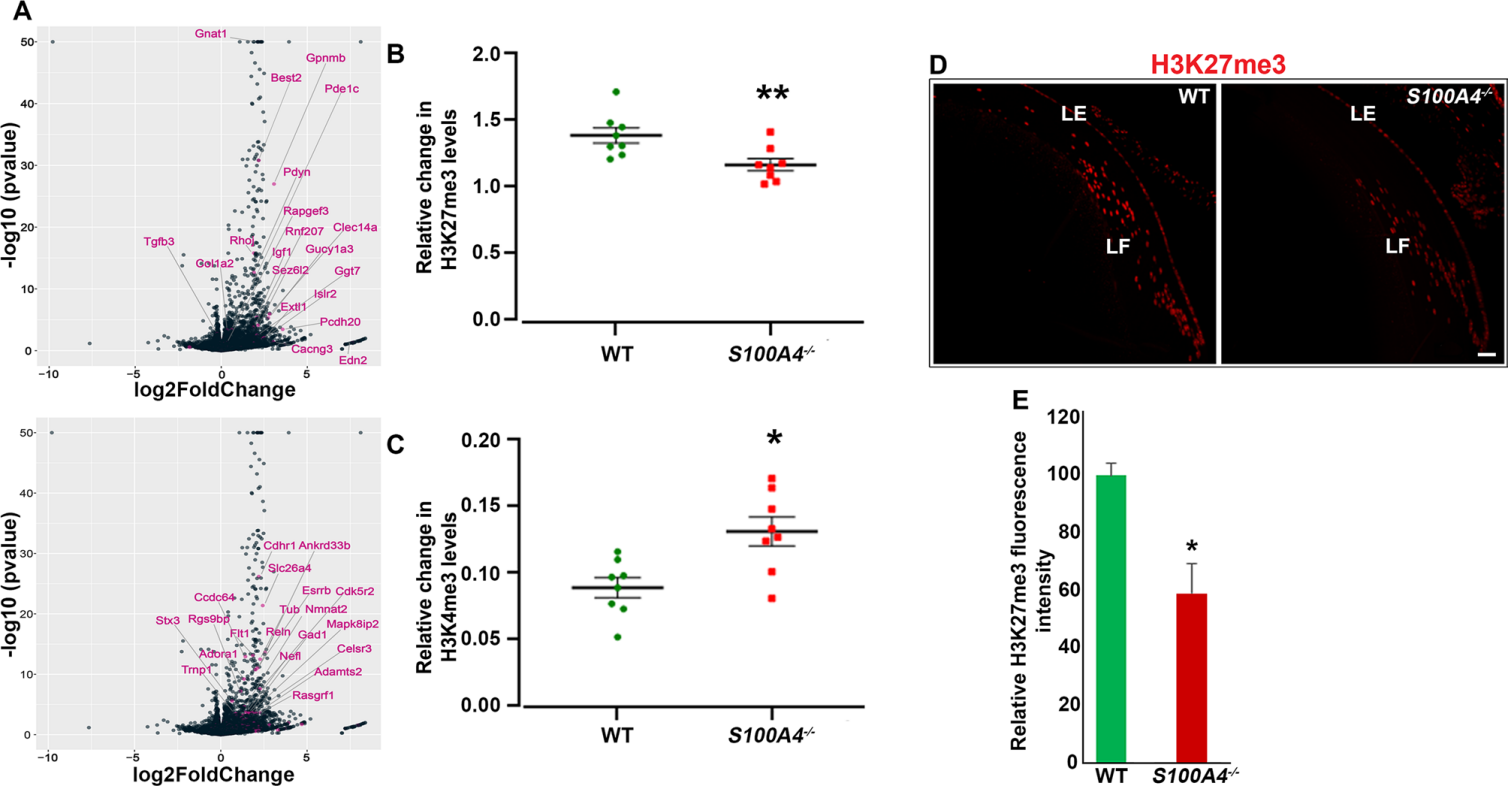
Association between S100A4 deletion-induced gene expression and H3K methylation in mouse lens. (**A**) Volcano plots depicting the differential expression results and highlighting genes with intermediate or high CpG-density promoters bearing histone trimethylation marks at K27 (H3K27me3) or K4 (H3K4me3) or at both (bivalent) in *S100A4*^−/−^ mouse lenses. (**B**,**C**) Significant changes (decrease and increase, respectively) in the levels of H3K27me3 and H3K4me3 in *S100A4*^−/−^ lenses compared to WT lenses based on ELISA analysis (*P < 0.05; **P < 0.01; Mann Whitney U test; n = 8, biological replicates). (**D**,**E**) Decreased staining for H3K27me3 in P30 *S100A4*^−/−^ lens compared to WT lens based on immunofluorescence analysis (*P < 0.05; n = 3, biological replicates). Scale bar 50 μm. *LE* lens epithelium, *LF* lens fibers.

Interestingly, based on gene set enrichment analysis, *S100A4*^−/−^ lenses upregulated a large number of genes whose promoters contain an intermediate or high density of CpG islands bearing trimethylation marks at histone-3 lysine 27 (H3K27), histone-3 lysine 4 (H3K4) and bivalent H3K27 and H3K4 (Fig. 3E). These histone methylation markings are known to be involved in determining cell state and lineage potential of embryonic stem cells, neural progenitor cells and embryonic fibroblasts^36,37^. Moreover, examination of individual genes within these gene sets identified many photoreceptor- and retina-specific genes including *Nrl*, *Neurod1*, *Six6*, *Rax*, *Reln*, *Gnat1*, *Best2*, *Rtbdn*, *Pde6b*, *Optc*, *Kcnb1*, *Slc24a1*, *Cacng3*, *Gucy1a2* and several others (Fig. 6A). Since trimethylation of H3K27 and H3K4 has been well-established to regulate gene suppression and activation, respectively^38,39^, we addressed whether S100A4 expression in the lens may affect H3K methylation. This was accomplished by quantitative ELISA-based analysis of trimethyl H3K27 and H3K4 levels in the total histone fraction extracted from *S100A4*^−/−^ and WT lenses at P30. While there was a significant decrease in the levels of H3K27me3 in the knockout (Fig. 6B), the levels of H3K4me3 increased significantly (Fig. 6C). Methylated H3K27 and H3K4 were normalized to total histone content in these analyses. The decrease in H3K27me3 was corroborated by immunostaining (Fig. 6D,E).

## Discussion

**Figure 7:**
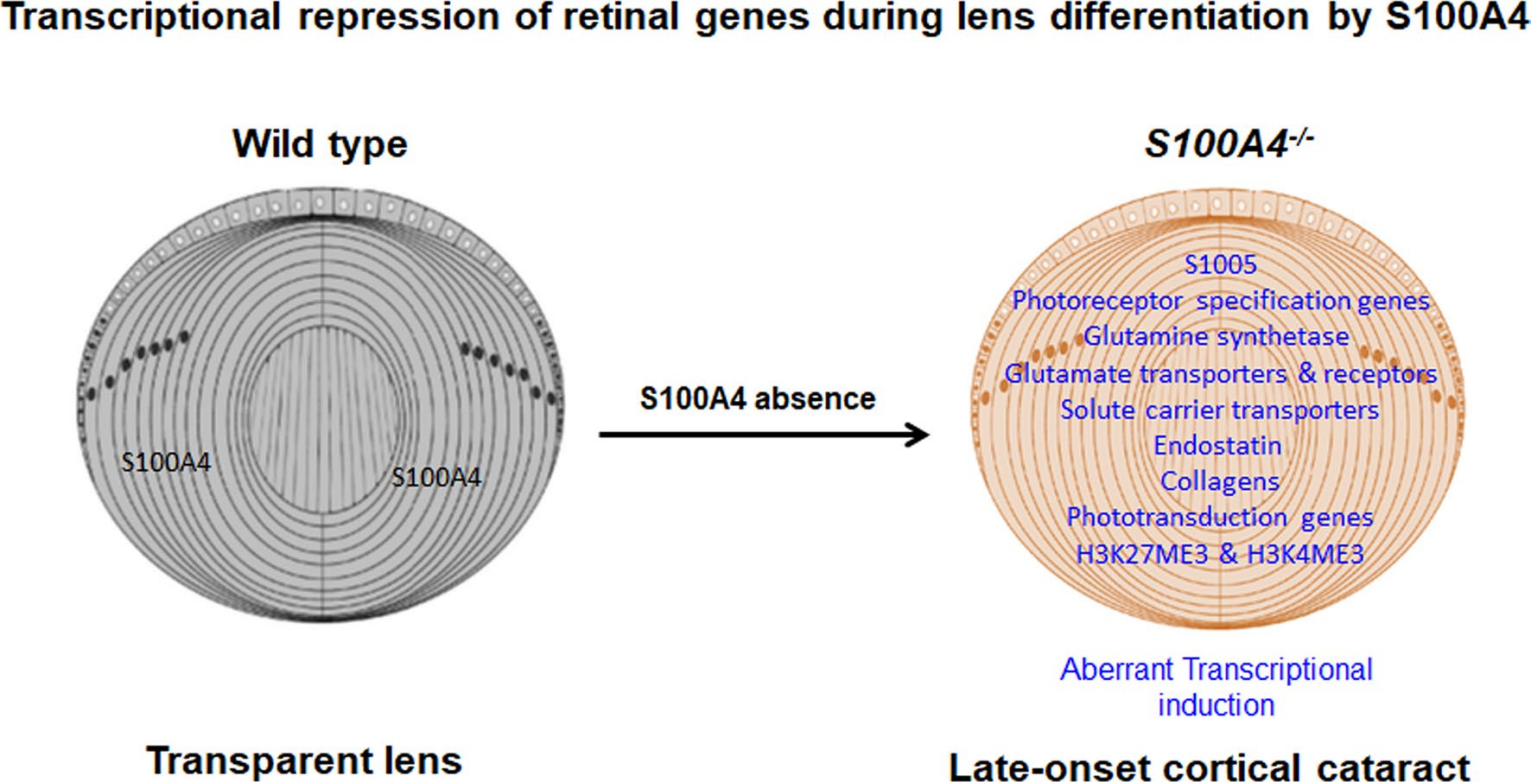
Graphical summarization of transcriptional repression of neurosensory genes during lens differentiation by S100A4. Absence of S100A4, a calcium binding protein expressed discretely in lens fibers within the mouse eye induces aberrant differentiation programs of non-lineage photoreceptor, Müller glia and olfactory sensory neurons in association with changes in histone-3 lysine (H3K) trimethylation markings, indicating a repressive role for S100A4 in suppressing expression of sensory neuron genes during lens differentiation. Lens drawings were adapted from the review article of Cvekl and Zhang (2017), and permission was obtained from Elsevier publisher.

In this study, we report a novel role for S100A4 in the suppression of non-lineage genes in the developing lens (Fig. 7). We demonstrated that S100A4 is specifically expressed in fiber cells of the lens and that the loss of S100A4 in the lens leads to an aberrant induction of genes involved in differentiation of the retina and eventual lens opacification. Importantly, the induction of retinal genes in the S100A4 knockout lens includes several retina- and photoreceptor-determining transcription factors. Thus, S100A4 appears to play a critical role in lens differentiation by repressing genes responsible for retinal specification at the point when the retina and the lens are separating in development.

This novel function of S100A4 in lens resembles its previously reported role to suppress the expression of osteoblast-specific genes and transcription factors in periodontal ligament cells^40^. Additionally, S100A4 regulates transcriptional programs during the development of mature microfold cells in Peyer’s patches^41^, suggesting that S100A4 may have a broad role in transcriptional regulation across many tissues. In this study, absence of S100A4 induced expression in the lens of several transcription factors including *Crx, Nrl, Rax,Otx2,Vsx2* and *Nr2e3,* other regulators (*Mab21l2*) and their target genes which have been demonstrated to be critical for the development and function of retina and photoreceptors^26,27,42^.

Our findings raise several mechanistic questions regarding S100A4 function. The first is how S100A4 suppresses transcription. Many genes whose expression is altered in *S100A4*^−/−^ lenses are enriched with promoters containing a medium-to-high density of CpG islands regulated by histone trimethylation, including H3K27me3 and H3K4me3 modifications. Consistently, these two histone trimethylation marks are altered in *S100A4*^−/−^ lenses, suggesting that abnormal transcription in the *S100A4*^−/−^ lens is potentially regulated by epigenetic mechanisms. Of note, changes in H3K27 and H3K4 trimethylation have been similarly reported to contribute to retinal development^43^. Similarly, histone modification regulated by the lysine acetyltransferases (CBP and p300), has been shown to play a crucial role in lens fate determination^44^. The second question concerns how epigenetic changes are induced by *S100A4* deletion. Expression of genes encoding histone and DNA methyltransferases and demethylases, protein components of the polycomb (PRC2) and/or trithorax complexes and nucleosomes, all of which are involved in regulating chromatin remodeling^45,46^ were not altered significantly in lenses lacking S100A4. Another intriguing question relates to how S100A4 functions in transcriptional repression, given its cytoplasmic localization in lens fibers. Although S100A4 has been found in the nuclei of other cells^3,10^, we did not observe the protein in the nuclei of lens fiber cells. Interestingly, S100A10, which belongs to the S100 family of proteins has been demonstrated to regulate breast cancer stem cell specification and pluripotency by regulating H3K27me3 chromatin marks^47^. While S100A5 is robustly upregulated in the absence of S100A4, it remains unclear as to what role (if any) S100A5 plays in the *S100A4*^−/−^ lens. An observation potentially relevant in this context is that S100A5 expression is upregulated or induced in response to a change from H3K4 dimethylation to trimethylation during embryonic stem cell differentiation^48^. Overall, there is little evidence for any functional redundancy among members of the S100 family^9,25^ despite their significant homology. Whether the robust upregulation of S100A5 expression starting from the embryonic stage in *S100A4*^−/−^ lenses has any role in induction of retinal genes needs to be addressed in future studies.

Interestingly, although the aberrant induction of retina and photoreceptor differentiation programs in lenses lacking S100A4 starts from the early neonatal developmental stages, this abnormality does not appear to, per se, overtly perturb lens morphogenesis and growth. However, it is likely that interactions between age-related stress factors and dysregulated expression of retinal and Müller glia genes, especially those engaged in solute carrier transport and encoding extracellular matrix proteins, secondarily impair lens clarity in older S100A4^−/−^ mice. The impairment of lens clarity likely results from osmotic imbalance and leads to the development of late onset cortical cataract under the absence of S100A4 (Fig. 2).

In conclusion, our study revealed a novel role of S100A4 in gene suppression and cell fate determination in the ocular lens. The goals of future studies are to elucidate this S100A4 function on the mechanistic level and to understand how the absence of S100A4 induces late-onset cortical cataracts.

## Methods

### Mice

All experiments using mice were carried out in accordance with the recommendations of the Guide for the Care and Use of Laboratory Animals of the National Institutes of Health. The protocol was approved by the Institutional Animal Care and Use Committee of the Duke University School of Medicine. This study was also carried out in compliance with the ARRIVE guidelines. The *S100A4* ^−/−^ (null) strain mouse used in this study was maintained on a C57BL/6 J background and has been thoroughly characterized previously^19^. Both WT (C57BL/6 J strain) and littermate WT mice were used as controls in the *S100A4*^−/−^ mouse studies. Both male and female mice were used in the described studies. Mice were maintained under a 12 h dark and light cycle with ad libitum food and water as we described earlier^49^. *S100A4*^−/−^ and littermate WT mouse genotyping was carried out using PCR analysis of tail DNA and the oligonucleotide primer sets (Supplementary Table S4) described previously^49^. At required gestational ages, pregnant dams were anesthetized with Euthasol (Virbac AH, Inc., Fort Worth, TX, USA) and fetuses were extracted by hysterectomy.

### Mouse eye and lens imaging

Images of cataractous and transparent lenses from live *S100A4*^−/−^ mice were captured using a Nikon D7500 Wi-Fi 4 K Digital SLR Camera. Dark illuminated dissected lenses of *S100A4*^−/−^ and WT mice were imaged using a Zeiss Stemi 2000-C stereomicroscope equipped with an AxioCam ERc 5S camera (Carl Zeiss Microscopy, LLC, White Plains, NY, USA).

### Histological analysis

Enucleated eyes from *S100A4*^−/−^ and littermate WT mice (8- to 9 month-old) were fixed in 3.7% buffered formalin for 48 h at room temperature. The fixed specimens were subsequently dehydrated, embedded in paraffin, and cut into 5 µm thick sections with a rotary microtome (Leica Biosystems, Buffalo Grove, IL, USA), prior to staining with hematoxylin and eosin as we described earlier^49^. Images were captured using a Zeiss Axio Imager equipped with a Hamamatsu Orca ER monochrome CCD camera (Carl Zeiss Microscopy, LLC).

### RNA extraction, library construction and RNA-seq analysis

For RNA extraction, P30 lenses derived from *S100A4*^−/−^ and littermate WT mice were stored in RNAlater at − 80 °C until extraction. Six to twelve lenses were pooled per sample for the WT1, WT2, *S100A4*^−/−^ 1 and 2 samples. RNA was isolated using the RNeasy Micro kit (Qiagen, Inc., Valencia, CA, USA) with a minor modification. Tissue homogenates prepared in RLT buffer containing β-mercaptoethanol were incubated with proteinase K solution (20 mg/ml for 15 min at room temperature) prior to following the extraction protocol provided in the manufacturer’s instructions.

RNA was quantified using a Qubit instrument (Thermo Fisher Scientific, Waltham, MA USA). RNA quality was analyzed on an Agilent TapeStation Model 2200 (Agilent Technologies, Santa Clara, CA, USA). All samples yielded RIN numbers > 7. Stranded mRNA-seq libraries were constructed using the Kapa kit (Kapa Biosystems, Inc, Wilmington, MA, USA) and following manufacturer's instructions. Libraries were indexed using a single 6 bp index allowing for multiple libraries to be pooled. RNA-Seq libraries were then normalized to a concentration of 10 nM, and pooled in equimolar ratio. The final pool of libraries was sequenced in one lane of an Illumina HiSeq 4000 sequencing flow cell (Illumina, Inc, San Diego, CA) with a 50 bp single end read length. Resultant raw bcl files were converted into fastq files and sequences demultiplexed using Illumina bcl2fastq conversion software version 2 (Illumina, Inc).

### RNA-seq transcriptome-based analysis of differential gene expression

RNA-seq raw data were processed using the Trim Galore toolkit which employs Cutadapt to trim low quality bases and Illumina sequencing adapters from the 3′ end of the reads. Only reads that were 20nt or longer after trimming were used for further analysis. Reads were mapped to the mouse genome and transcriptome (GRCm38v73 version) using the STAR RNA-seq alignment tool^50,51^. Gene counts were compiled using the HTSeq tool for reads that mapped to a single genomic locus. Only those genes that had at least 10 reads in any given library were used in subsequent analysis. Normalization and analysis of differential expression was carried out using the DESeq2, Bioconductor package with the R statistical programming environment^52,53^. We considered a gene differentially expressed if exhibits a greater than or equal to two-fold difference in expression between the *S100A4*^−/−^ and littermate controls, and an adjusted P value of less than or equal to 0.05. Gene Set Enrichment Analysis (GSEA)^54^ was performed to identify differentially regulated pathways and gene ontology terms. The false discovery rate (FDR) was used to correct for multiple hypothesis testing, and pathways were considered significant if the FDR ≤ 5%. Network analysis was performed using the Qiagen Ingenuity Pathway Analysis (IPA) tool to identify upstream regulators of differentially regulated genes in *S100A4*^−/−^ mouse lenses.

### qRT-PCR

To confirm the results of RNA-seq based identification of DEGs in *S100A4*^−/−^ lenses, qRT-PCR analysis was performed for selected DEGs. For this, RNA was extracted from pooled P1 or P30 *S100A4*^−/−^ and littermate WT mouse lenses as described above using an RNeasy Micro kit and reverse transcribed using the Advantage RT for PCR Kit (Clontech Laboratories, Inc., Mountain View, CA, USA). Reverse-transcribed single-stranded cDNA and gene-specific forward and reverse oligonucleotide PCR primers (Table S4) were used for qRT-PCR analyses as we described previously^49^. A few of the selected gene transcripts were also amplified by RT-PCR analyses and the amplified DNA products were separated on an agarose gel and visualized with GelRed Nucleic Acid Stain (Biotium, Hayward, CA, USA) and imaged using Chemidoc Touch (Bio-Rad, Hercules, CA, USA). The amplified DNA products were excised, purified using a gel extraction kit (Qiagen, Inc., Valencia, CA, USA) and sequenced to confirm gene identity.

### Histone extraction and histone-3K trimethylation analysis

The EpiQuik Total Histone Extraction Kit (Epigentek Group Inc. Farmingdale, NY) was used to extract total histone from P30 *S100A4*^−/−^ and littermate WT lenses as per manufacturer’s instructions. Total histone protein isolated from the *S100A4*^−/−^ and littermate WT lenses were used to quantify H3K27 and H3K4 global trimethylation using EpiQuik Global H3K27 and H3K4 Tri-Methylation Assay Kits (Epigentek Group Inc.). Briefly, the levels of histone proteins tri-methylated at K27 or K4 were quantified by colorimetric analysis at 450 nm using high-affinity antibodies specific to trimethylated H3K27 or H3K4 in conjunction with horse radish peroxidase conjugated secondary antibody and a SpectraMax M5 microplate reader (Molecular Devices LLC. San Jose, CA, USA).

### Measurements of intracellular calcium in lens

Lens intracellular calcium levels were measured as we described earlier^21^. Briefly, lenses dissected from P60 *S100A4*^−/−^ and littermate WT mice were placed in a Sylgard Petri dish containing normal Tyrode solution (137.7 mM NaCl, 2.3 mM NaOH, 5.4 mM KCl, 2 mM CaCl_2_, 1 mM MgCl_2_, 5 mM Hepes, and 10 mM glucose, pH 7.4.). A small volume of 2 mM FURA2 solution was injected into the fiber cells at different depths in the lens. FURA2 was dissolved in the pipette solution containing 83 mM K-aspartic acid, 17 mM KCl, 10 mM NaCH_3_OSO_3_, and 5 mM Hepes, and pH adjusted to 6.9 with KOH. Images were acquired and the ratios of fluorescence emission calculated from 360 and 380 nm excitation curves. The ratios were converted into [Ca^2+^]i using the [Ca^2+^] calibration curve corresponding to the appropriate lens depth as described previously^21^.

### Measurements of gap junction-coupling conductance

Gap junction coupling conductance was measured as we previously described^55^. Briefly, one microelectrode was placed in a central fiber cell into which a wide-band stochastic current was injected. The induced voltage was recorded by a second microelectrode that was placed in a peripheral fiber cell r (cm) from the center of a lens. The impedance (induced voltage ÷ injected current) of the lens was recorded in real time using a fast Fourier analyzer (Hewlett Packard, Palo Alto, CA). The high frequency asymptote of the magnitude of the impedance is proportional to gap junction coupling resistance. The voltage recording microelectrode was advanced from the surface to the center of the lens, recording coupling at five or six locations. Data from 5 or 6 lenses were pooled to obtain the final resistance curve.

### Tissue homogenates and immunoblotting

#### Tissue homogenates

Lens epithelium and fiber mass samples derived from P21 mice and lenses derived from P30 *S100A4*^−/−^ and littermate WT mice were homogenized at 4 °C using a glass homogenizer and ice cold hypotonic buffer (10 mM Tris buffer, pH 7.4, containing 0.2 mM MgCl2, 5 mM N-ethylmaleimide, 2.0 mM sodium orthovanadate, 10 mM sodium fluoride, 60 μM phenylmethylsulfonyl fluoride, 0.4 mM iodoacetamide, protease inhibitor cocktail and PhosSTOP phosphatase inhibitor cocktail (one each/10 ml buffer; obtained from Roche, Manheim, Germany)). Tissue lysates were spun at 800 g for 10 min at 4 °C, and the protein content of the supernatant measured using a Micro BCA Protein assay kit (Thermo Fisher Scientific). For the separation of lens cytosolic (100,000 g supernatant) and membrane-enriched fractions (100,000 g pellet), the 800 g lysates prepared above were subjected to ultracentrifugation by spinning at 100,000 g for 1 h at 4 °C. Pellets were suspended in 8 M urea buffer, and protein was estimated using Pierce 660 nm protein assay reagent (Thermo Fisher Scientific).

Lens fiber cytosolic and nuclear fraction proteins of P30 WT mice were extracted using nuclear fraction extraction kit from BioVision (BioVision, Inc, Milpitas, CA, USA) by following the manufacturer’s protocol. Briefly, freshly extracted lenses were dissected to remove the capsule/epithelium and the fiber mass was used. The protein content of cytosolic and nuclear fractions was determined using BCA Protein Assay Reagent Kit. GAPDH and Histone H2B antibodies were used to confirm the purity of cytosolic and nuclear protein fractions, respectively.

For preparation of retinal lysates, mouse eyes were dissected along the Ora serrata to remove and discard the anterior chamber along with lens. The eyecup was further washed with cold PBS to detach the retina, and retinas homogenized in RIPA buffer (Sigma-Aldrich, St. Louis, MO, USA) containing both protease and phosphatase inhibitors prior to determining protein content using Pierce BCA protein Assay Kit (Thermo Fisher Scientific).

#### Immunoblotting

Equal amounts of protein were resolved by sodium dodecyl sulfate–polyacrylamide gel electrophoresis (SDS–PAGE, using an appropriate percent of acrylamide depending on the test protein analyzed), followed by electrophoretic transfer to nitrocellulose membrane. The membranes were subsequently blocked for 2 h in 5% blocking grade milk protein in TBST buffer (Tris buffered saline containing 0.1% Tween 20) followed by incubation with primary antibodies (details are provided in Supplementary Table S5) overnight at 4 °C. After washing thoroughly, blots were incubated with appropriate secondary antibodies for 2 h at room temperature and developed by enhanced chemiluminescence using Chemidoc Touch system (Bio-Rad). Immunostained protein bands were quantified using ImageJ Software. For normalizing protein loading for membrane-rich proteins, protein fractions were resolved on SDS-PAGE, stained with Gel code blue protein stain (Thermo Fisher Scientific), and destained with distilled water prior to densitometric quantification using ImageJ software. Protein bands detected at 37 kDa and 15 kDa were used for normalization. For analysis of soluble proteins, GAPDH was immunoblotted as a loading control using anti-GAPDH antibody after stripping the respective blots.

### Tissue processing, immunofluorescence and imaging

Tissue specimens from WT mice (heads of E12.5; E13.5; E14.5 and E17.5 and eyes of postnatal (P21), and *S100A4*^−/−^ and littermate WT mice (head of E16.5 and eyes of P30) were fixed for cryosectioning in 4% buffered paraformaldehyde for 24 h at 4 °C, transferred into 5% and 30% sucrose in PBS (Phosphate-buffered saline) on consecutive days as we described previously^49^. After embedding in optimal cutting temperature media (Tissue-Tek, Torrance, CA, USA), samples were cut into 10 μm-thick sagittal sections using a Microm HM550 Cryostat (GMI, Ramsey, MN, USA) and stored at − 80 °C. Additionally, P30 mouse eyes were cut at the sagittal and equatorial plane. Air-dried tissue cryosections were treated with Image-iT FX signal enhancer (Invitrogen, Eugene, OR, USA) and blocked in blocking buffer (5% globulin-free bovine serum albumin and 5% filtered goat serum in 0.3% Triton X-100 containing PBS) for 30 min each. Tissue sections were then incubated overnight at 4 °C with primary antibodies (details in Supplementary Table S5). After washing, sections were incubated with appropriate Alexa Fluor (488 or 594) –conjugated secondary antibodies for 2 h at room temperature. For F-actin staining, preblocked equatorial sections were labeled with tetra rhodamine isothiocyanate–conjugated phalloidin (TRITC; Sigma Aldrich) for 2 h at a 1:500 dilution, washed, and mounted as described above. All representative immunofluorescence data reported in this study are based on analysis of a minimum of four tissue sections derived from four independent specimens (biological replicates). Images were captured using an Eclipse 90i confocal laser scanning microscope (Nikon Instruments, Inc., Melville, NY, USA).

For paraffin sectioning and immunohistochemistry, P30 day-old eyes from *S100A4*^−/−^ and littermate WT mice were fixed and sectioned as we described previously^56^. Paraffin sections were deparaffinized with three changes of Xylene and hydrated with absolute alcohol and antigen retrieval was performed as we described earlier^56^. Tissue sections were blocked for 10 min in a humidified chamber with medical Background Sniper reducing solution (Biocare Medical, Concord, CA, USA), prior to incubation for 24 h at 4 °C with H3K27ME3 or S100A4 rabbit polyclonal antibodies. Tissue sections were washed in TBS (Tris-buffered saline) buffer and incubated with Alexa Fluor 594 and 488–conjugated secondary antibodies (Invitrogen, Grand Island, NY, USA; at a 1: 200 dilution) in a dark humidified chamber for 2 h at room temperature. After this, sections were washed again with Tris buffered saline buffer, mounted on slides using Vectamount and nail polish, then imaged using a Nikon Eclipse 90i confocal laser scanning microscope to obtain single optical images.

For staining retinal sections, P30 *S100A4*^−/−^ and littermate WT eyes were enucleated and fixed for 30 min with 4% paraformaldehyde in PBS buffer, pH 7.5. The anterior chambers along with lenses were removed, and the eyecups were further fixed overnight at 4 °C. The following day, eyecups were rinsed three times for 5 min each with 1X PBS, and embedded in 4% agarose (Invitrogen). One hundred micron-thick sections from the central portion of the retina were collected into 24 well plates using a vibratome (HM 650 V, Microm). The floating sections were then incubated on an orbital shaker for 4 h with blocking solution containing 3% donkey serum and 0.1% Triton X-100 in PBS buffer. These sections were further incubated overnight with either S100A4 or S100A5 rabbit polyclonal antibodies, washed three times with PBS, and incubated for 2 h with goat anti-rabbit Alexa Fluor 488 secondary antibody (1:500). After thoroughly washing with PBS for several times, sections were stained with Hoechst (1:1000) for 15 min. Following a final wash with PBS, sections were mounted under coverslips using Fluoromount G (Electron Microscopy Sciences, Hatfield, PA), then imaged using a Nikon Eclipse 90i confocal microscope.

### Statistical analysis

Statistical analyses were performed using GraphPad Prism version 7. Mann Whitney U test was used to compare 2 groups. Data from immunoblot quantification experiments and qRT-PCR were analyzed by the Student’s t-test, a P < 0.05 was considered to define statistically significant differences between *S100A4*^−/−^ and littermate controls. Values are presented as mean ± standard deviation (SD) for qRT-PCR data and mean ± standard error of the mean (S.E.M) for immunoblots. All the immunoblots were representative of 3–5 biological replicates.

## Supplementary Information

Supplementary Information 1.

Supplementary Information 2.

Supplementary Information 3.

Supplementary Information 4.

## Data Availability

All raw RNA-seq data and normalized expression values are available in the Gene Expression Omnibus with accession number- GSE143909. All other data of this study are included in the article and supplementary information files.

## Abbreviations

BCA: Bicinchoninic acid assay
DEGs: Differentially expressed genes
ELISA: Enzyme-linked immunosorbent assay
FDR: False discovery rate
GAPDH: Glyceraldehyde 3-phosphate dehydrogenase
GSEA: Gene set enrichment analysis
H3K: Histone 3 lysine
IPA: Ingenuity pathway analysis
IRB: Institutional review board
KEGG: Kyoto encyclopedia of genes and genomes
LC: Loading control
LE: Lens epithelium
LF: Lens fiber
LV: Lens vesicle
MSigDB: Molecular signatures database
PC: Principle component
RT-PCR: Reverse transcription polymerase chain reaction
qRT-PCR: Quantitative reverse transcription polymerase chain reaction
RU: Relative units
SDS-PAGE: Sodium dodecyl sulfate–polyacrylamide gel electrophoresis
TRITC: Tetramethylrhodamine isothiocyanate
WT: Wild type
