# Supplementary material for: Absence of S100A4 in the mouse lens induces an aberrant retina-specific differentiation program and cataract
Source: Sci Rep. 2021 Jan 26;11:2203. doi: 10.1038/s41598-021-81611-y (PMC7838418; doi:10.1038/s41598-021-81611-y)
Supplement: Supplementary file 1 — Supplementary Information 1. [file 41598_2021_81611_MOESM1_ESM.pdf]

Absence of S100A4 in the mouse lens induces an aberrant retina-specific differentiation program and cataract

Rupalatha Maddala[1*], Junyuan Gao[2], Richard T. Mathias[2], Tylor R. Lewis[1], Vadim Y. Arshavsky[1, 3], Adrian Levine[4], Jonathan M Backer[4, 5], Anne R. Bresnick[4], and Ponugoti V. Rao[1, 3]*

Fig. S1

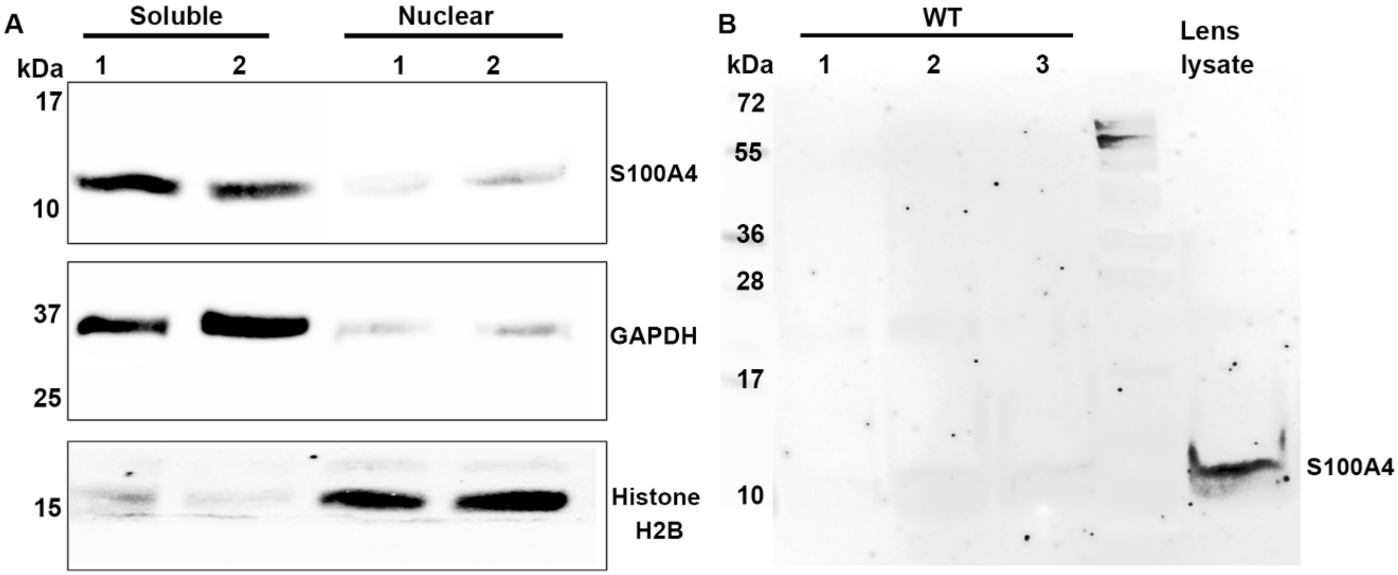
A
Soluble
Nuclear
kDa
1
2
1
2
17
10
S100A4
37
25
GAPDH
15
Histone
H2B
B
WT
Lens
lysate
kDa
1
2
3
72
55
36
28
17
10
S100A4

Fig. S2

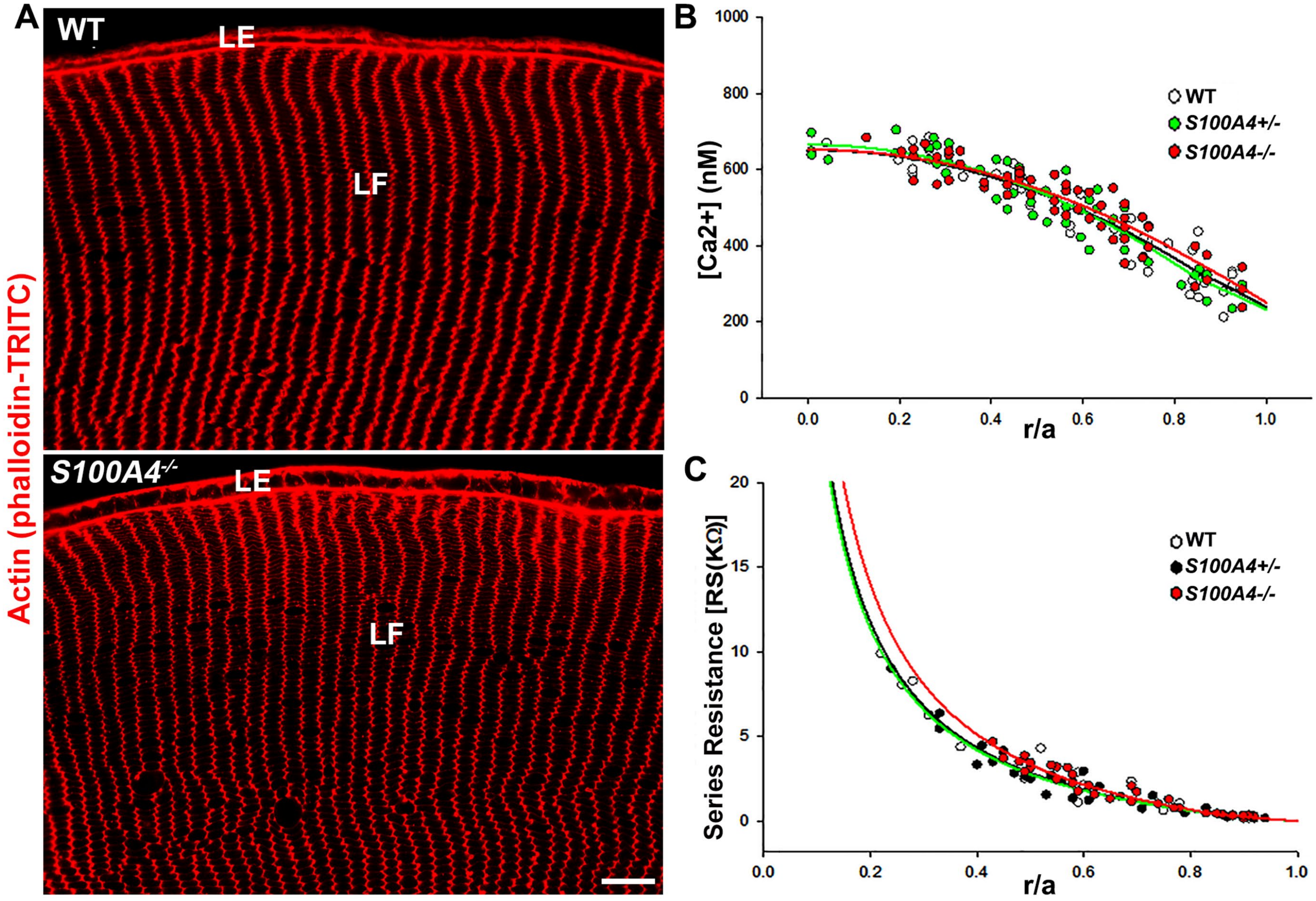
A
WT
LE
LF
S100A4-/-
LE
LF
Actin (phalloidin-TRITC)
B
[Ca2+] (nM)
1000
800
600
400
200
0
0.0
0.2
0.4
0.6
0.8
1.0
r/a
WT
S100A4+/-
S100A4-/-
C
Series Resistance [RS(KΩ)]
20
15
10
5
0
0.0
0.2
0.4
0.6
0.8
1.0
r/a
WT
S100A4+/-
S100A4-/-

Fig. S3

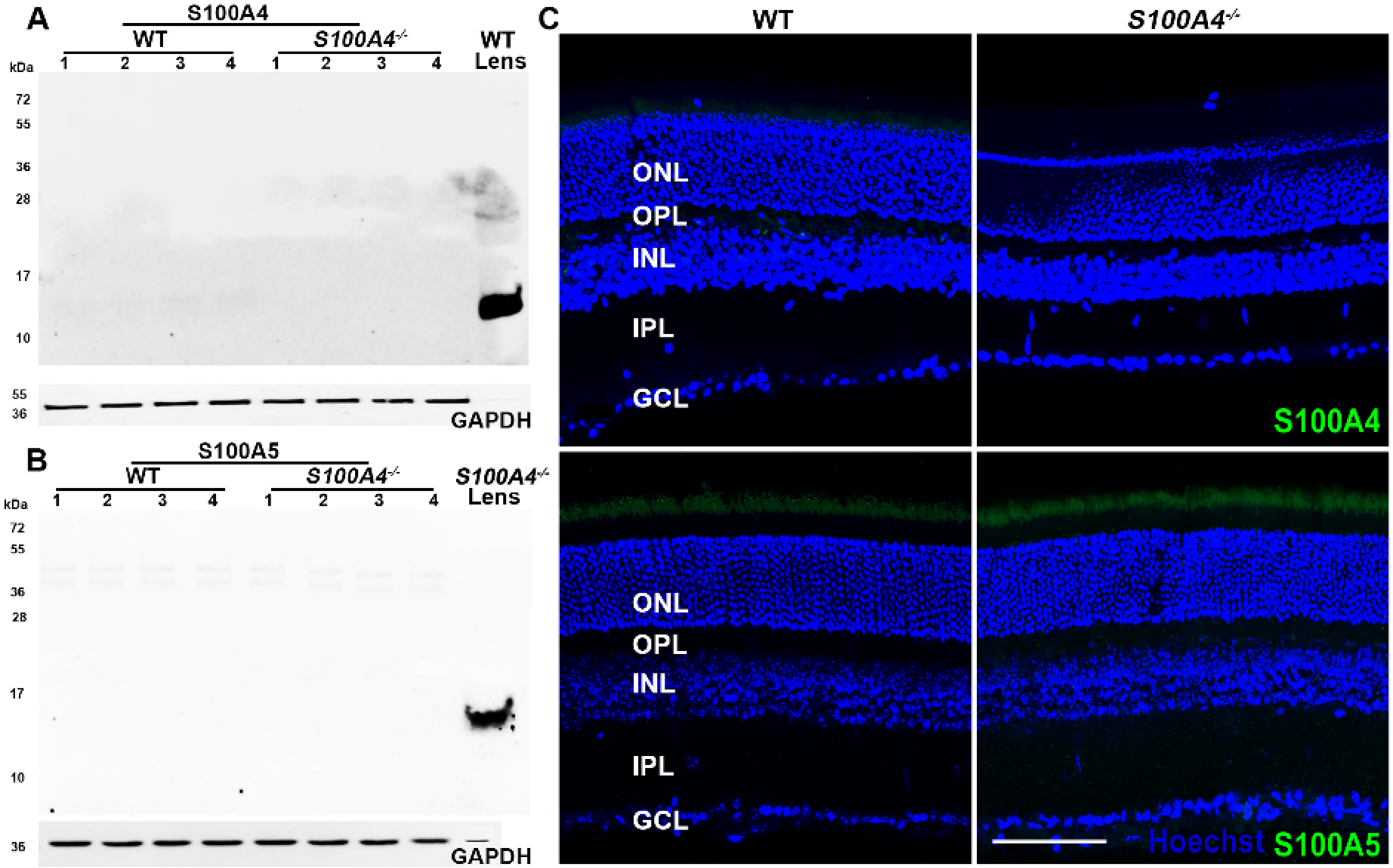

A
S100A4
WT
S100A4-/-
1
2
3
4
1
2
3
4
WT Lens
kDa
72
55
36
28
17
10
55
36
GAPDH
B
S100A5
WT
S100A4-/-
1
2
3
4
1
2
3
4
S100A4-/- Lens
kDa
72
55
36
28
17
10
36
GAPDH
C
WT
S100A4-/-
ONL
OPL
INL
IPL
GCL
S100A4
ONL
OPL
INL
IPL
GCL
Hoechst S100A5

Fig. S4

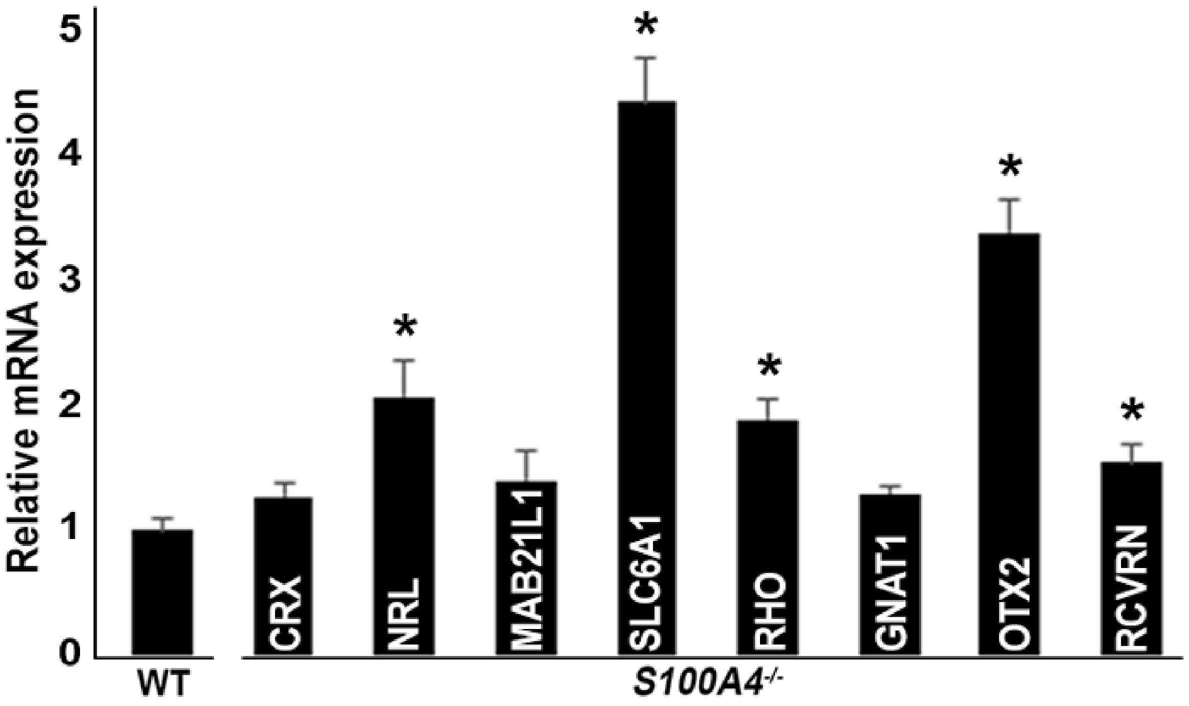

Relative mRNA expression
5
4
3
2
1
0
*
*
*
*
*
CRX
NRL
MAB21L1
SLC6A1
RHO
GNAT1
OTX2
RCVRN
WT
S100A4-/-

Fig. S5

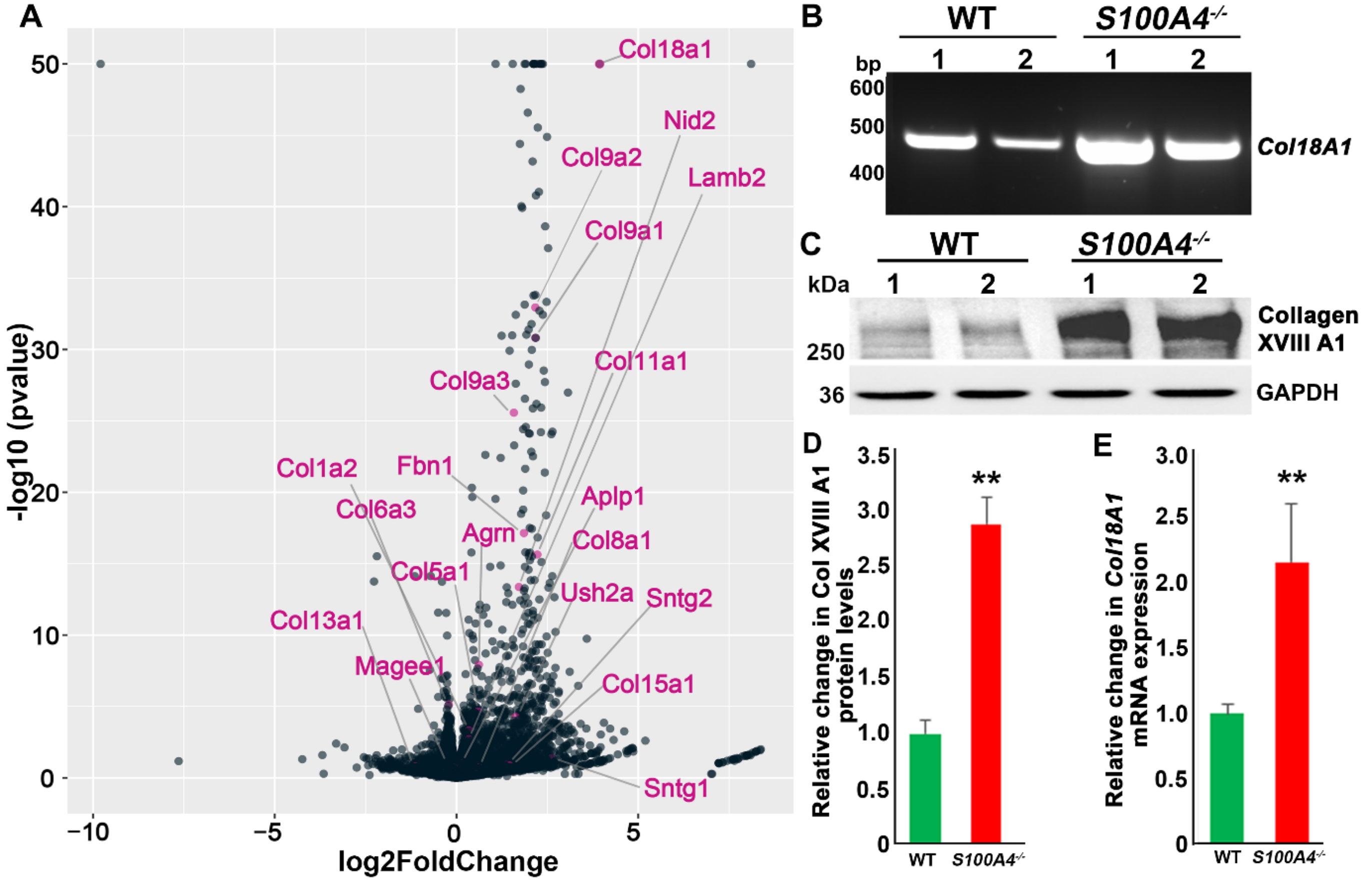
A
B
C
D
E
-log10 (pvalue)
log2FoldChange
Col18a1
Nid2
Col9a2
Lamb2
Col9a1
Col11a1
Col9a3
Col1a2
Fbn1
Aplp1
Col6a3
Agrn
Col8a1
Col5a1
Ush2a
Sntg2
Col13a1
Magee1
Col15a1
Sntg1
WT
S100A4-/-
bp
1
2
600
500
400
Col18A1
kDa
250
36
Collagen XVIII A1
GAPDH
Relative change in Col XVIII A1 protein levels
**
Relative change in Col18A1 mRNA expression

Fig. S6

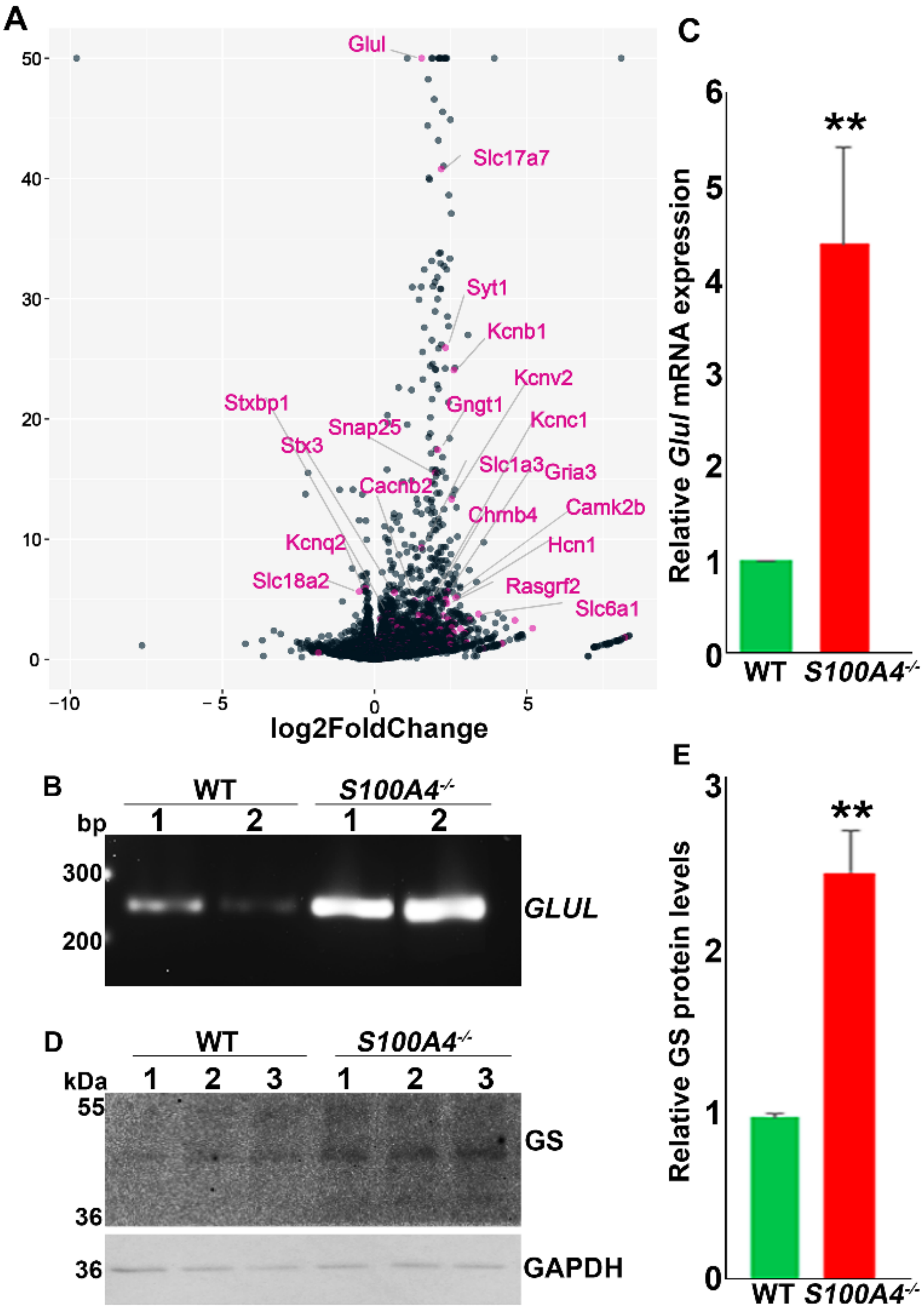
A
Glul
Slc17a7
Syt1
Kcnb1
Kcnv2
Gngt1
Kcnc1
Stxbp1
Snap25
Stx3
Slc1a3
Gria3
Cacnb2
Chrnb4
Camk2b
Kcnq2
Hcn1
Slc18a2
Rasgrf2
Slc6a1
log2FoldChange
B
WT
S100A4-/-
bp
300
200
GLUL
C
Relative Glul mRNA expression
**
WT
S100A4-/-
D
kDa
55
36
GS
GAPDH
E
Relative GS protein levels
**

Fig. S7

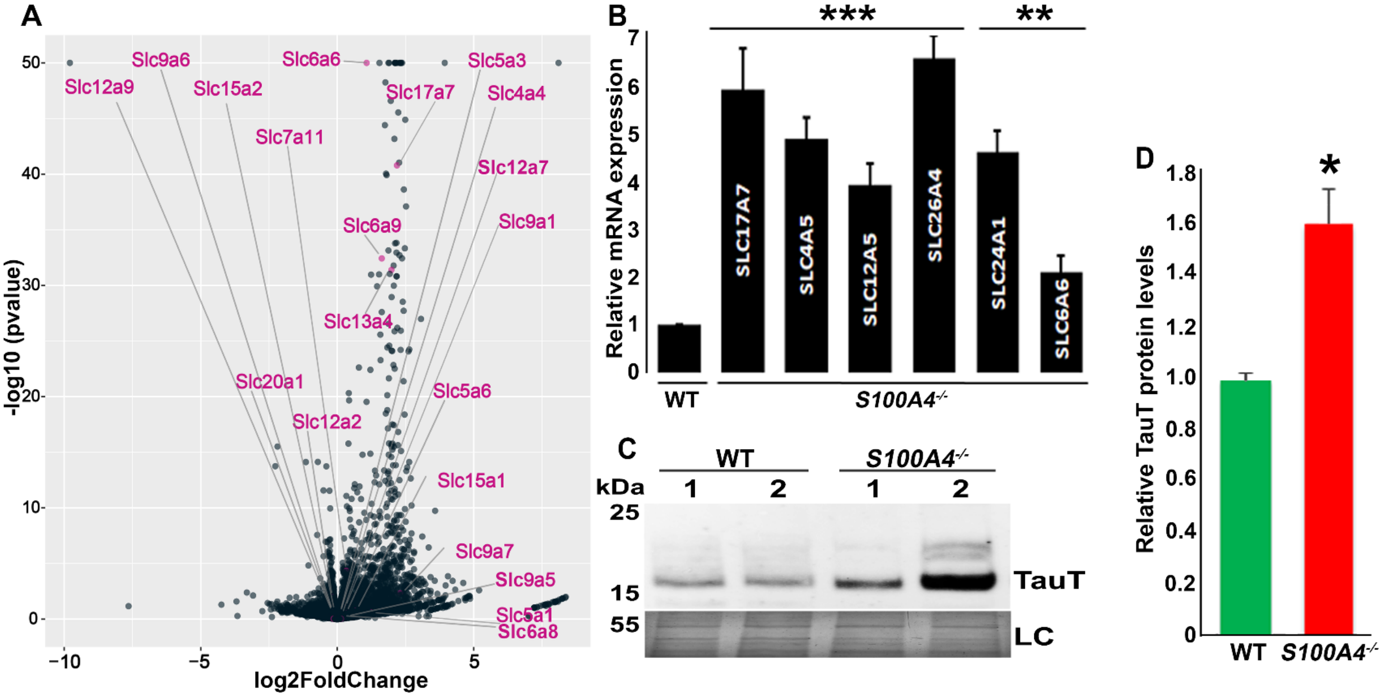
A
Slc12a9
Slc9a6
Slc15a2
Slc7a11
Slc6a6
Slc17a7
Slc5a3
Slc4a4
Slc12a7
Slc9a1
Slc6a9
Slc13a4
Slc20a1
Slc12a2
Slc5a6
Slc15a1
Slc9a7
Slc9a5
Slc5a1
Slc6a8
-log10 (pvalue)
log2FoldChange
B
Relative mRNA expression
SLC17A7
SLC4A5
SLC12A5
SLC26A4
SLC24A1
SLC6A6
WT
S100A4-/-
C
WT
S100A4-/-
kDa
25
15
55
TauT
LC
D
Relative TauT protein levels
WT
S100A4-/-

Table S2. Upregulated and downregulated genes in *S100A4*$^{-/-}$ mouse lenses by the criteria of fold-change ≥ 2 and adjusted p-value of ≤ 0.05.

	Diras2	*Tppp*	*Lhx2*	*Itm2a*	*Nrxn2*	*Col9a3*	*Pde1a*
**Upregulated ⬆**	*Ccdc141*	*Sv2b*	*Rhoj*	*Ckmt1*	*Chrna6*	*Fbln1*	*Tceal6*
	Dlg2	*Lrtm1*	*Sgk1*	*Plcxd2*	*Asns*	*Rax*	*Lcn2*
Scara5	*Rims2*	*Dnm1*	*Slc24a1*	*Pdyn*	*Adamtsl1*	*Unc5c*	*Rundc3a*
Rnf207	*Syn1*	*Cnga1*	*Nrl*	*Cabp4*	*Mfap4*	*Prrt1*	*Fam13a*
Tyr	*Rasef*	*Pdc*	*Tulp1*	*Adhfe1*	*A730017C20Rik*	*Khdrbs3*	*Lynx1*
Rtbdn	*Mak*	*Rdh12*	*Snap25*	*Crx*	*Wdr17*	*Cplx1*	*Ism2*
Gpr152	*Nr2f1*	*42795*	*Clec18a*	*Tnfrsf1b*	*Guca1a*	*Adora1*	*Rgs9*
Igf2	*Plagl1*	*Atp2b2*	*Boc*	*Vtn*	*Nid2*	*Kcng4*	*Gpr116*
Thbd	*Nkd2*	*Slc17a7*	*Impg1*	*Psd*	*Kcnc1*	*3010026O09Rik*	*Chi3l1*
Pde2a	*Pitpnm3*	*Syt1*	*Meg3*	*Asic4*	*Slc7a1*	*Pde1a*	*Rassf2*
Wnk4	*Lrp2*	*Pde6b*	*Tmem150c*	*Fam57b*	*Bmp2*	*Tceal6*	*Chgb*
Impg2	*Calb2*	*Cacna2d2*	*Zic5*	*Esrrb*	*Cdk5r2*	*Lcn2*	*Kcnj13*
Cldn1	*Ldhb*	*Lrit2*	*Unc80*	*Il33*	*Camk2b*	*Rundc3a*	*Bcam*
Ankrd9	*Samd11*	*Kdr*	*Cygb*	*Ptk2b*	*Pde6g*	*Fam13a*	*Slc17a8*
Cacna1f	*Kcnj9*	*Cacnb2*	*Nefl*	*Kcnv2*	*Adamts9*	*Lynx1*	*Iqsec3*
Sparcl1	*Ppp1r1b*	*Cacna2d4*	*Nrxn3*	*Mpp4*	*Mfrp*	*Ism2*	*Slc6a9*
Hbb−b1	*Fbln5*	*BC027072*	*Slc5a5*	*Disp2*	*Sez6l2*	*Rgs9*	*Cntn1*
Lrrc38	*Slco4a1*	*Emp1*	*Srcin1*	*Slc38a8*	*Syp*	*Gpr116*	*Tnfrsf21*
Pltp	*Frem1*	*Vcan*	*2610034M16Rik*	*Tmem132c*	*Fhdc1*	*Chi3l1*	*Epas1*
Atp2b4	*Lrp5*	*Col9a2*	*Rorb*	*Gm16119*	*Bambi*	*Rassf2*	*Fmod*
Postn	*Sgip1*	*Grk1*	*Nxnl1*	*Gria3*	*Ocln*	*Chgb*	*Cgn*
Arrdc2	*Mmd2*	*Rp1l1*	*Plxnc1*	*Fam129a*	*Slc6a13*	*Kcnj13*	*Rcvrn*
F13a1	*Bcat1*	*Cdhr1*	*Acsl6*	*Cngb1*	*Otx1*	*Bcam*	*Best2*
Slc4a10	*Rgs9bp*	*Gpr165*	*Rs1*	*Plch2*	*Bsn*	*Slc17a8*	*Klhdc8a*
Clec14a	*Gucy2f*	*Papss2*	*Zfp385b*	*Rgs7bp*	*Pcdh20*	*Iqsec3*	*Vsx2*
Wfikkn2	*Hcn1*	*Fam107a*	*Mrc2*	*Kcnb1*	*Kcnj3*	*Slc6a9*	*Sorcs1*
Muc4	*Rasgrf2*	*Tgfbr2*	*Hsd17b11*	*Tub*	*Ust*	*Cntn1*	*Nr2e3*
Bmp4	*Pcdh15*	*Cdh20*	*Deptor*	*Chrnb4*	*Cadps*	*Tnfrsf21*	*Amigo2*
Rims3	*Ssc5d*	*Rbp3*	*Acot11*	*Chga*	*Dach1*	*Epas1*	
Sh3gl2	*Nrp2*	*Ush2a*	*Cadm4*	*Gucy2e*	*Plekhb1*	*Fmod*	
Sult4a1	*Slc1a3*	*Rho*	*Pianp*	*St8sia1*	*Igfn1*	*Cgn*	**Downregulated ⬇**
Notch3	*Eln*	*Rom1*	*Apcdd1*	*Neurod1*	*AL591207.1*	*Flt1*	
Sncb	*Mcf2l*	*Tmem59l*	*Nap1l5*	*Six6*	*Slc22a8*	*Col23a1*	*S100a4*
Cacna1g	*Gucy1a3*	*Camkv*	*Pag1*	*Gpnmb*	*Cldn19*	*Napb*	*Dynlt1b*
Pld5	*Csf1r*	*Col2a1*	*Otx2*	*Cplx4*	*Slc4a5*	*Cldn2*	*Cck*
Cobll1	*Adamts2*	*Atp1a3*	*4930594M22Rik*	*Mlana*	*Rasgrf1*	*Atp1b2*	*Pttg1*
Slc16a2	*Gsta3*	*F5*	*Tnfaip3*	*Sod3*	*Penk*	*Optc*	*Rab4a*
Mapk8ip2	*Gad1*	*Col18a1*	*Cplx3*	*Chac1*	*Gckr*	*Ltbp2*	*Gstt1*
Pmel	*Serpina3n*	*Ucp2*	*AI847159*	*Guca1b*	*Prph2*	*Rarres2*	
Mef2c	*Col11a1*	*Gm21743*	*Rd3*	*Lactbl1*	*Slc13a4*	*Apoe*	
Rab3c	*Tenm1*	*Ntrk2*	*Gpr56*	*S100a5*	*Prelp*	*Atp1a2*	
Dsg2	*Shisa2*	*Slc12a5*	*Sema3e*	*Ccdc64*	*Flt1*	*Slc6a6*	
Scg2	*Fzd8*	*Ifit3*	*Shisa6*	*Glul*	*Col23a1*	*Reln*	
Thy1	*Fbn2*	*Scrt1*	*Matn2*	*Sag*	*Napb*	*Notum*	
Axl	*mmu−mir−3078*	*Iqgap2*	*Ankrd33*	*Gnat1*	*Cldn2*	*Prom1*	
Mab21l2	*Celf4*	*Ankrd33b*	*Sphkap*	*Dct*	*Atp1b2*	*Tbx2*	
Frmpd4	*Aipl1*	*Clic6*	*Tyrp1*	*1700112E06Rik*	*Optc*	*Nmnat2*	
Cadm3	*Celsr3*	*Edn3*	*Lphn3*	*Efemp1*	*Ltbp2*	*Col9a3*	
Rasgrf1	*Gpm6a*	*Gm14290*	*Fbln2*	*Necab2*	*Rarres2*	*Fbln1*	
Penk	*Snhg11*	*Col9a1*	*Esrrg*	*Plin4*	*Apoe*	*Rax*	
Gckr	*Zic2*	*Slc26a4*	*Prex2*	*Crhbp*	*Atp1a2*	*Unc5c*	
Prph2	*Rlbp1*	*Abca8a*	*Abcg1*	*Mlph*	*Slc6a6*	*Prrt1*	
Slc13a4	*Rp1*	*Unc5b*	*Dio3*	*Gngt1*	*Reln*	*Khdrbs3*	
Prelp	*Lrit1*	*Fbn1*	*Tfap2b*	*Gpx3*	*Notum*	*Cplx1*	
Slc6a1	*Zic1*	*Elavl3*	*Pdlim3*	*Pde6a*	*Prom1*	*Adora1*	
Gjd2	*Synpr*	*Wfdc1*	*Prdm8*	*Igfbp4*	*Tbx2*	*Kcng4*	
	Lrrn2	*Ctsh*	*Igf1*	*Igfbp2*	*Nmnat2*	*3010026O09Rik*	

Table S4. Oligonucleotide primers used for qRT-PCR and genotyping analyses:

qRT-PCR

Accession	Gene Name	Forward Primer	Reverse Primer
NM_011311.2	S100A4	GAGGAGGCCCTGGATGTA	GCTCCTTGAGCTCTGTCTTG
NM_011312.2	S100A5	TTCAGGGAGAGAGGGTAGC	GCTGCTCTCCTTCATCTTCTC
XM_006505861	RHO	CAGAAGGCAGAGAAGGAAGTC	CTGGTGGGTGAAGATGTAGAAG
NM_008140	GNAT1	GACGACGAAGTGAACCGAAT	GTTGAGGAAGAGCACGATAGAC
NM_011881	GRK1	GCTGAAGGAAGGGCAGAATAA	GGCAAAGTAGTCCACAGAGAAG
XM_006525880	PDE6a	GGCTACCGGAGAATCACTTAC	GTGAAGTACCGTTTCAGCTTTC
NM_012065	PDE6g	GGTGATAGGAGGACCAGTCA	CCCTTGCACGCCTTTCT
XM_017313271	SLC24A1	AGAGGAGGAAGATGAGGAAGAG	GGCAGGAGGAAGAGGTAGAT
XM_006503709	CNGA1	GGTGGCAGATGACGGAATTA	AGCCTTGCTGCCTTTGATA
NM_001165934	RGS9	GGTGTCTCTTGGAGGAATTGT	AACTGGGTGTCGTCTGTTATC
XM_006523462	GUCA1B	CACATCCCTGCTACTGATACAC	GCCTTCACCAGCCAATCT
NM_001313971	RDH12	CTTCACTCGAGAACTGGCTAAG	GCAAACACAGCAGGTAGGA
NM_001159730	PDC	CAGACAAATGTCCTCTCCTCAG	GCACTGCCTCCGGTATTT
NM_001271916	NRL	GTTGGGCTCCACACCATAC	GCCAGCCAATATAGCTCCTC
NM_009073	ROM1	TGGTGGGCTGAAAGACATAC	CTTGGGAGGTTCTTCATCTGG
NM_009038	RCVRC	GTGCTGGAGATCGTCATGG	GATCTTCTCAGCCCGCTTT
NM_008806	PDE6b	CTATACTGTCCGGGCCTATCT	GTTCGTGGGCCTGAGTATG
NM_001113330	CRX	GGCTGTCCCATACTCAAGTG	GCATACACATCCGGGTACTG
NM_053245	AIPL1	GGAGGTTGAGTGGCTGAAG	CTGGGTGGTGTCGTAGAATG
NM_013833	RAX	CCCAAGGAGCAAGGAGAAG	AGCTCGTGCAGTTGGTAAG
NM_013708	NR2E3	GGATGTGCCCAGTGGATAAG	CGAGGTTGGCGCTCATT
NM_008938	PRPH2	CCAGAAGAAGCGGGTCAAG	CCAAGCTGAAGAGGACGATG
NM_054095.2	NeCab2	CCCTACTCCTCCCTACATCC	CCAGCCCATCCTCCTTTG
XM_006513174	Col18a1	CACCCTCTCACTTGCTCATAC	AGGAGGCCTGACCTGTAG
NM_008131	Glul	GGTTTGGAATGGAGCAGG	TTCCAGATAGGACCCTGTGAG
NM_013708	NR2E3	GGATGTGCCCAGTGGATAAG	CGAGGTTGGCGCTCATT
NM_001290646	LHX2	CGTCCATCAGCAGTGACC	CAGCAGAGCCTCGAAGTG
NM_182993.2	SLC17a7	GGTCTTTGCTTCGGGAGAG	CAGCCTCGTCCTCCATTTC
NM_001166067.1	SLC4a5	GGCTCTGTGCTCCTTCAC	TGGTCTCCGTCTCCATCTT
NM_144813.1	SLC24a1	TCCTCCCAGCAGCCTATTA	GCATCCCATCCTCCACTTC
NM_011867.3	SLC26a4	CCGAACTCCCGGTGAAAG	CGCAATGACCTCACTCCTAC
NM_020333.2	SLC12a5	CGGGCAGAGGAGTCTATCA	GGTGGGACACACCATCAC
NM_009320	SLC6A6	ACCCAGGCAGTCTGAAATG	GGGAAAGTAGCGGTGAAGTAG
GU214026	GAPDH	GGAGAAACCTGCCAAGTATGA	CCTCAGTGTAGCCCAAGATG
NM_001286481	OTX2	GAGGGAAGAGGTGGCACTGA	GACGCTGGGCTCCAGATAGA
NM_011839.4	MAB21L2	CTCAGGCCAAGCTGGTTTA	TCCCATCTGGTTGAGGTAGA
NM_178703	SLC6A1	CATGTCACCAAGAGGTCCATAG	GTTAGACAGGCCAATCAGGTAG

Genotyping

Accession	Gene Name	Forward Primer	Reverse Primer
20198	S100A4	AGCTGGGGTTTTTCCACTTT	ATCCAACCCTTCATGGACAG

Table S5. Details of primary and secondary antibodies used for immunoblotting (IB) and immunofluorescence (IF) analyses

Antibodies	Cat. No.	Source	Dilution (IB)	Dilution (IF)
FSP1/S100A4 Rabbit Polyclonal Ab	07-2274	Millipore Sigma Life Science Center, Burlington, MA	1:1000	1:250
S100A5 Rabbit Polyclonal Ab	17924-1-AP	Proteintech Group, Inc, Rosemont, IL	1:1000	1:250
PDE γ-subunit rabbit antibody		Gift from R. H. Cote (University of New Hampshire, Durham, NH).	1:1000	1:250
Peripherin rabbit polyclonal antibody	PA3-16723	Thermo Fisher Scientific	1:1000	
GRK1 mouse monoclonal antibody [D11]	ab2776	Abcam.com, Cambridge, MA	1:1000	
Rhodopsin Mouse monoclonal antibody	ab98887	Abcam.com, Cambridge, MA	1:1000	
GNGα1 (2G11) Goat polyclonal	c-20	Santa Cruz Biotechnology, Inc. Dallas, TX	1:1000	
Transducin Gαt rabbit polyclonal Antibody	SC-389	Santa Cruz Biotechnology, Inc. Dallas, TX		1:250
Glutamine Synthetase mouse monoclonal ab	610517	BD Transduction Laboratories™ San Jose, CA	1:1000	
Collagen XVIIIA1 rabbit polyclonal antibody		Gift from Takako Sasaki PhD, OITA University, Japan.	1:2000	
GAPDH mouse monoclonal ab	60004-1	Proteintech Group, Chicago, IL	1:8000	
Histone H2B	8135S	Cell Signaling Technology, Danvers, MA	1:3000	
Hoechst 33258, pentahydrate (bis-benzimide)	H21491	Molecular Probes, Inc. / Thermo Fisher Scientific. Eugene, OR		1:5000
Phalloidin–Tetramethyl rhodamine B isothiocyanate (TRITC)	P1951	Sigma/Aldrich, St. Louis, MO		1:500
Tri-Methyl-Histone H3 (Lys27) (C36B11), rabbit monoclonal Ab	9733	Cell Signaling Technology, Danvers, MA		1:500
SLC6A6 Rabbit polyclonal antibody	A14783	ABclonal Technology, Woburn, MA	1:1000	

Secondary antibodies for immunofluorescence analyses:

Antibodies	Cat. No.	Source	Dilution
Alexa Fluor™ 488 goat anti-Rabbit IgG	A11077	Invitrogen / Thermo Fisher Scientific. Rockford, IL,	1:400
Alexa Fluor™ 568 goat anti-Rabbit IgG	A11004	Invitrogen / Thermo Fisher Scientific. Rockford, IL,	1:200
Alexa Fluor™ 568 rabbit anti-goat IgG	A11079	Invitrogen / Thermo Fisher Scientific. Rockford, IL,	1:200
