# Supplementary material for: Absence of S100A4 in the mouse lens induces an aberrant retina-specific differentiation program and cataract
Source: Sci Rep. 2021 Jan 26;11:2203. doi: 10.1038/s41598-021-81611-y (PMC7838418; doi:10.1038/s41598-021-81611-y)

Absence of S100A4 in the mouse lens induces an aberrant retina-specific differentiation program and cataract

Rupalatha Maddala[1*], Junyuan Gao [2], Richard T. Mathias [2], Tylor R. Lewis[1],Vadim Y. Arshavsky[1, 3], Adrian Levine[4], Jonathan M Backer[4, 5], Anne R. Bresnick [4], and Ponugoti V. Rao[1, 3]*

Original immunoblot images used in the manuscript

Fig. 1B

S100A4 in lens Fiber mass and Epithelium

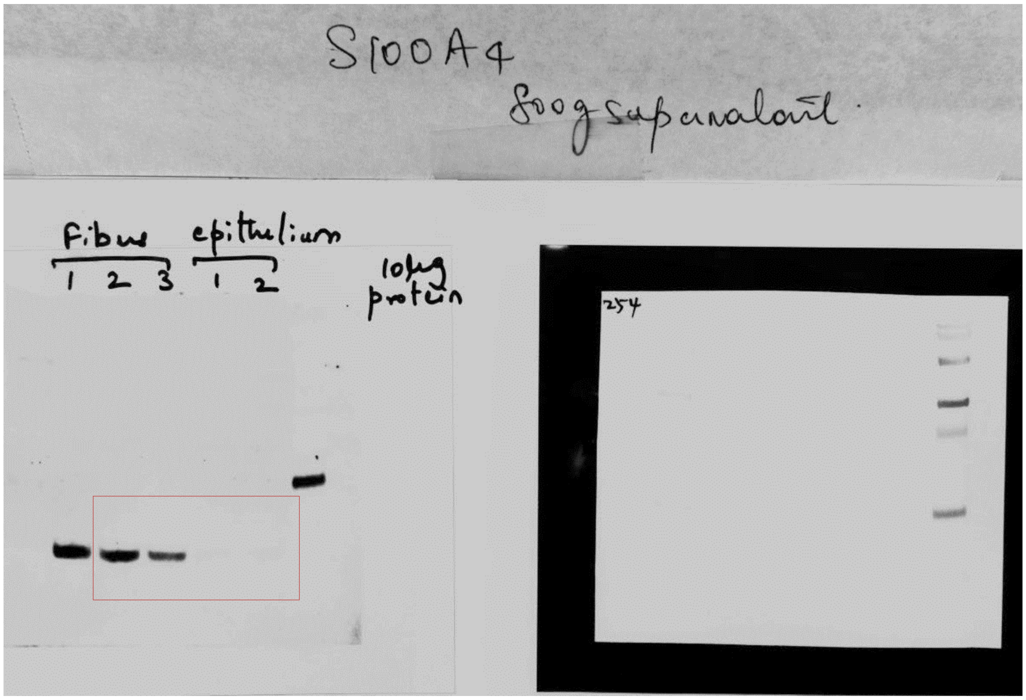

GAPDH in lens Fiber mass and Epithelium

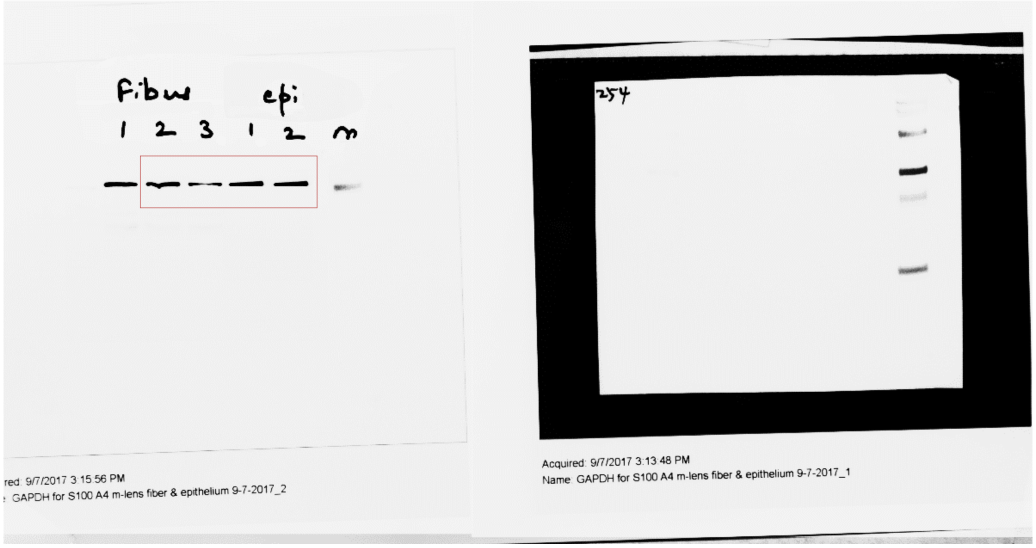

Fig. 1C

S100A4 in soluble and membrane fractions

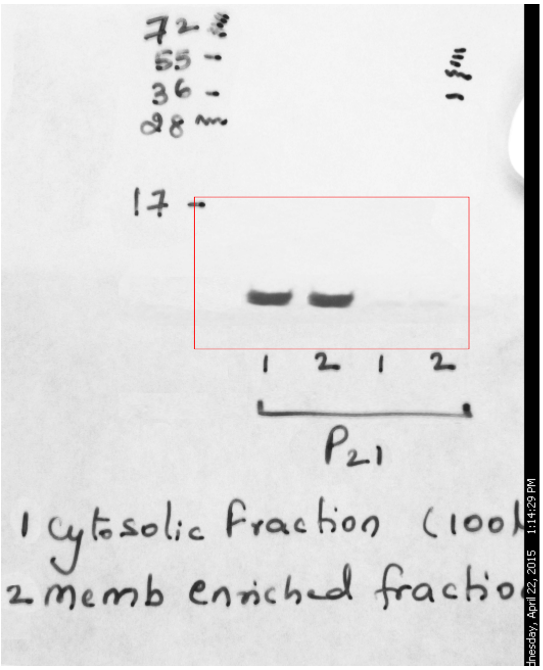

Loading controls for soluble and membrane fractions

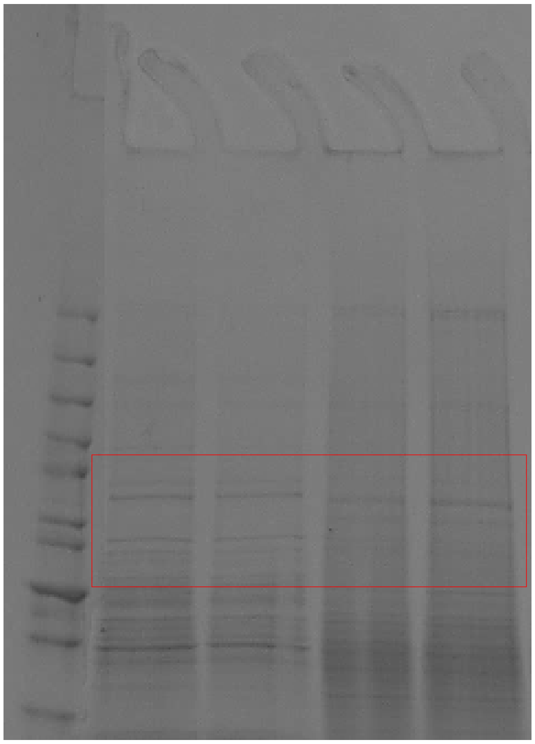

Fig. 4B

S100A5 and GAPDH in P30 *S100A4*$^{-/-}$ and WT lenses

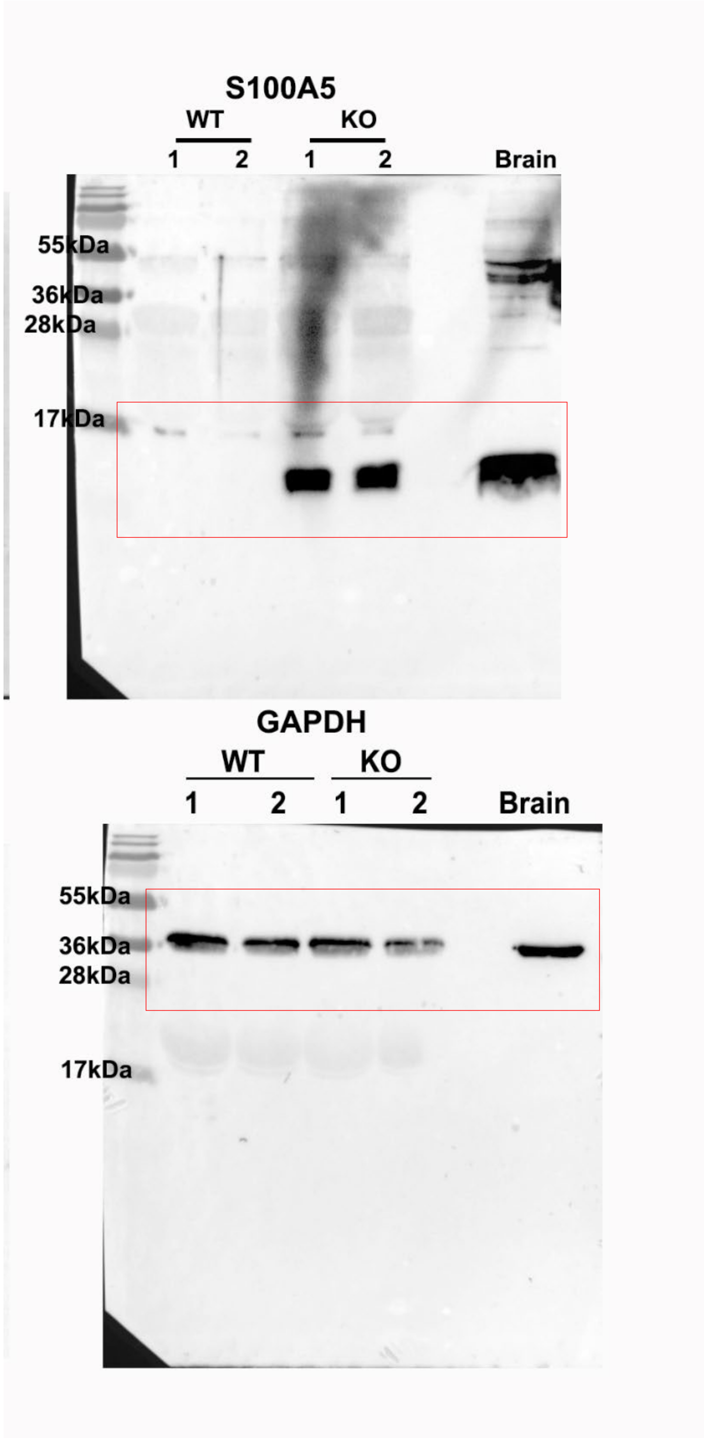

Fig. 5D

Photoreceptor specific proteins in *S100A4*$^{-/-}$ lens lysates

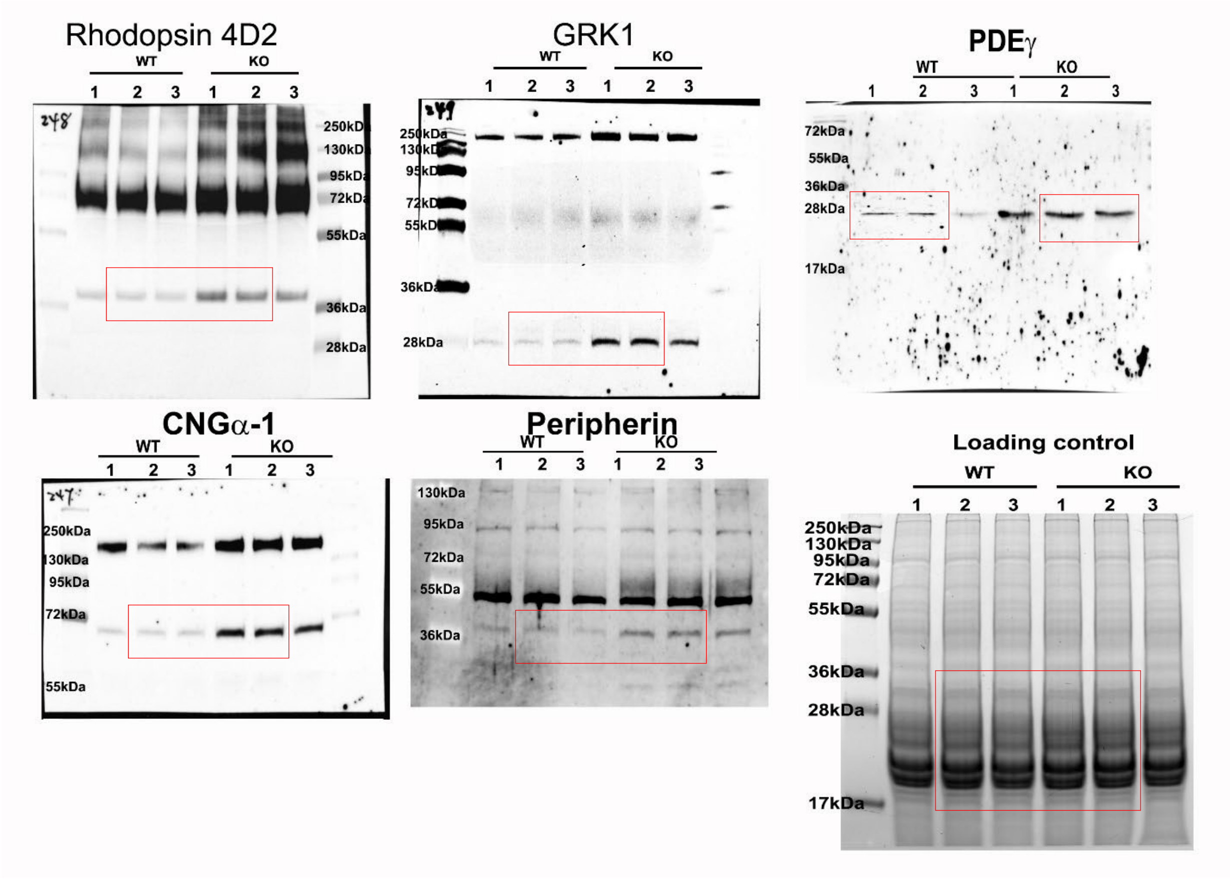

Fig. S1A

S100A4 was not detected in the nuclear fraction of the mouse lens fibers

S100A4 **GAPDH** **Histone2B**

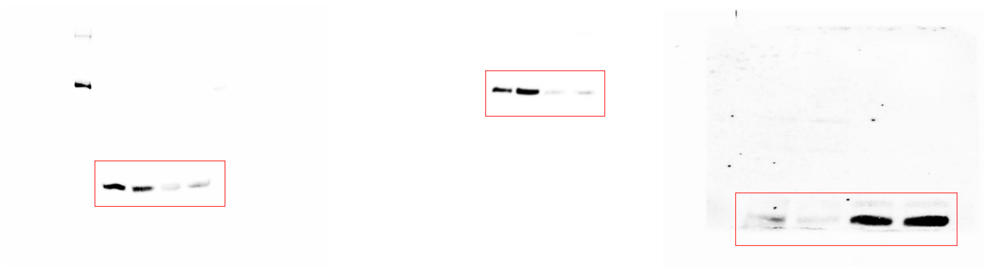

Fig. S1B

S100A4 was not detected in the conditioned medium of mouse lens.

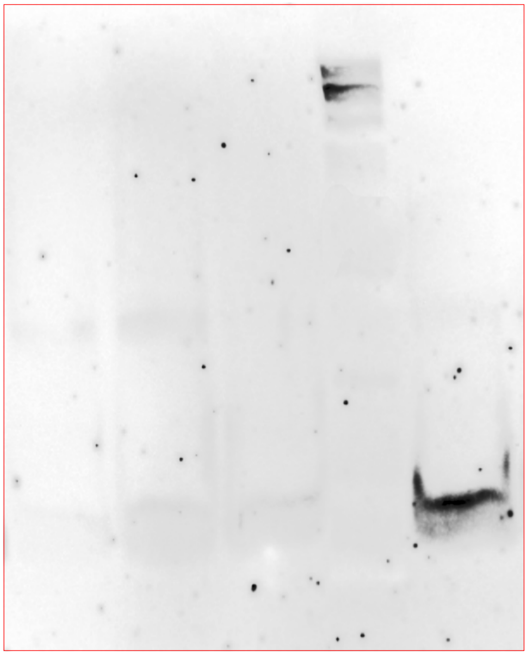

Fig. S3A

Wild type mouse retina does not express S100A4

S100A4 GAPDH

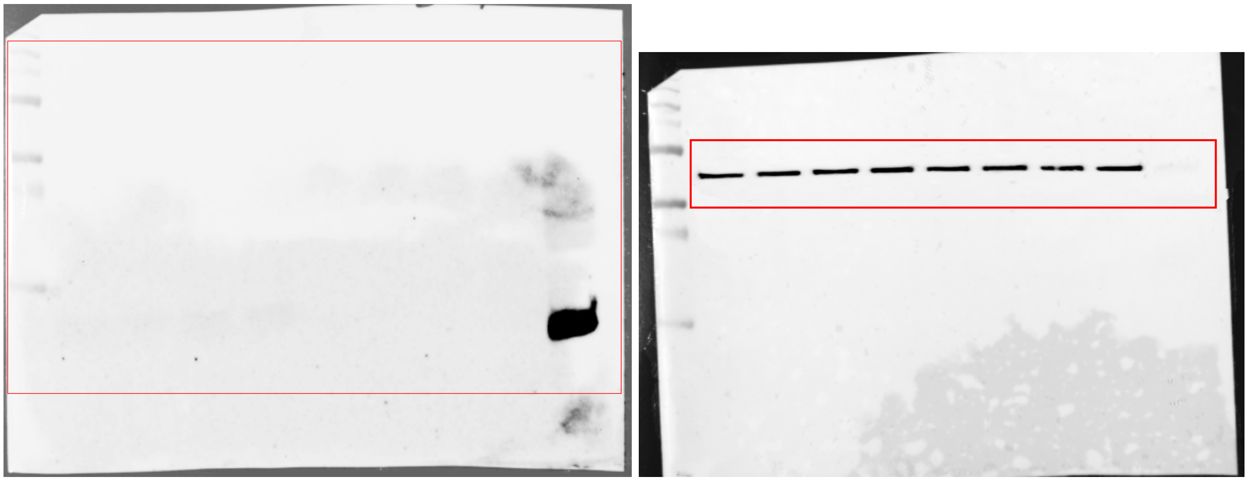

Fig. S3B

Wild type mouse retina does not express S100A5

S100A4 GAPDH

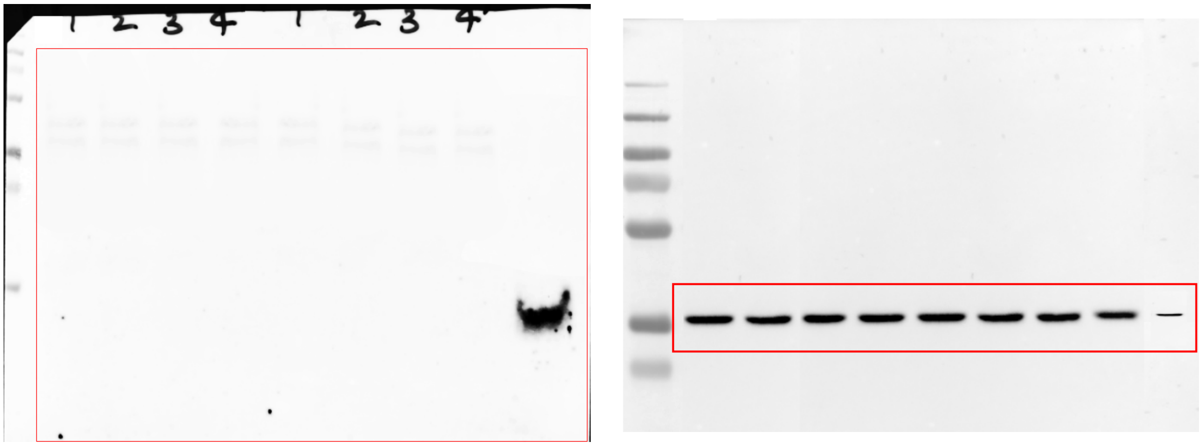

Fig. S5C

Upregulation of Collagen XVIII A1 in *S100A4* $^{-/-}$ mouse lenses.

Collagen XVIII A1

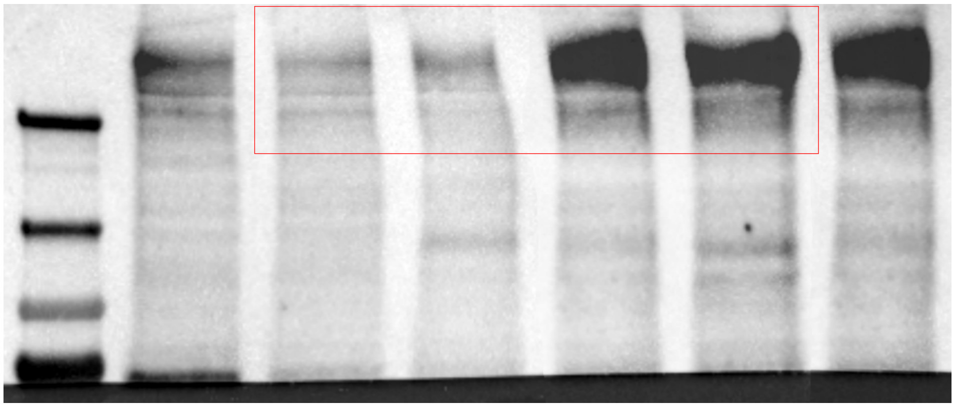

GAPDH

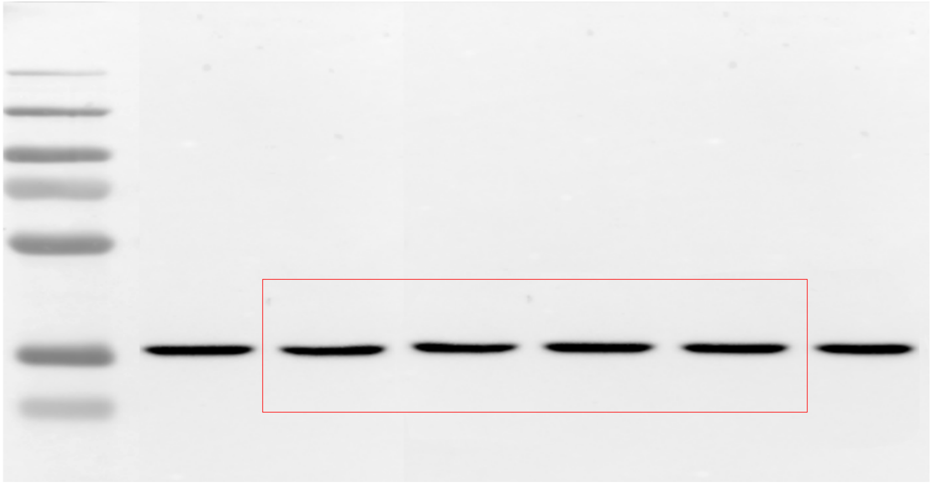

Fig. S6D

Induction of Glutamine Synthetase in *S100A4* $^{-/-}$ mouse lens

Glutamine Synthetase

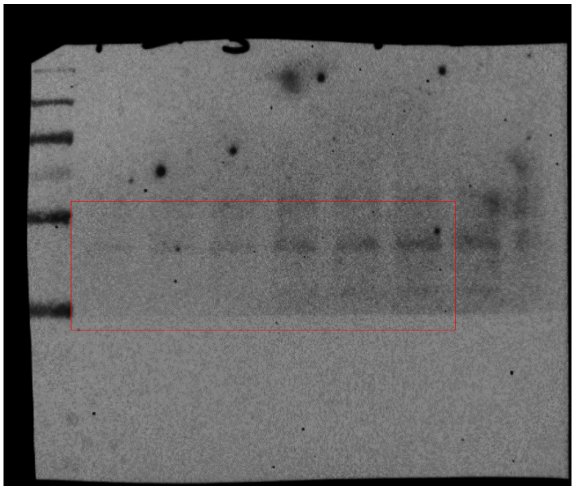

GAPDH

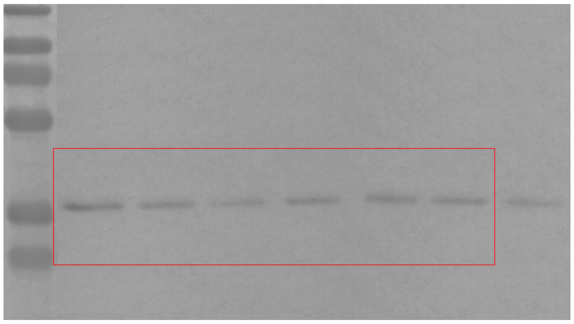

Fig. S7C

Upregulation of TauT protein in *S100A4*$^{-/-}$ lenses

TauT

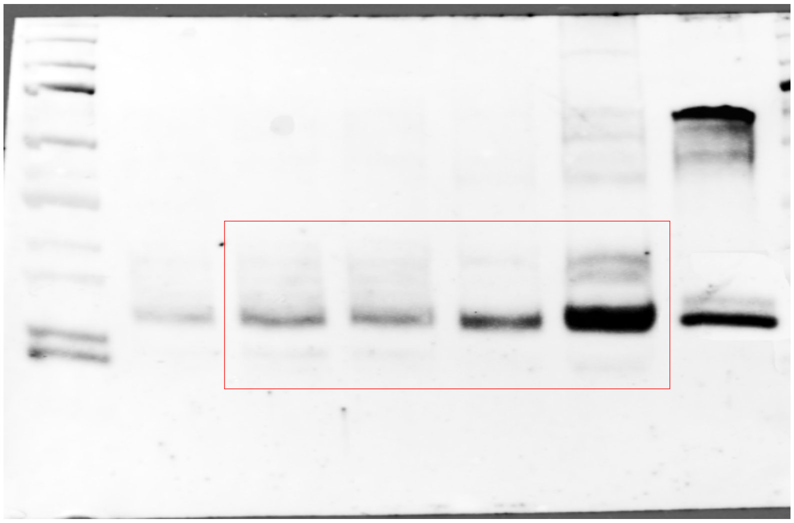

Loading Control

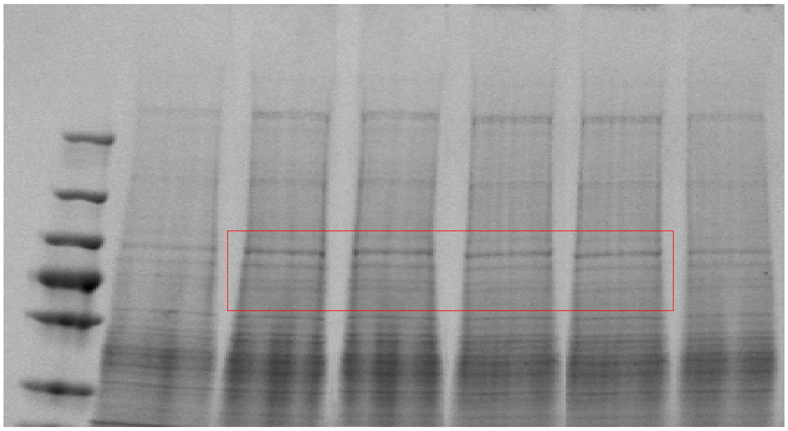

Supplement: Supplementary file 4 — Supplementary Information 4. [file 41598_2021_81611_MOESM4_ESM.pdf]
